# Many-Body Methods for Surface Chemistry Come of Age: Achieving Consensus with Experiments

**DOI:** 10.1021/jacs.3c09616

**Published:** 2023-11-10

**Authors:** Benjamin X. Shi, Andrea Zen, Venkat Kapil, Péter R. Nagy, Andreas Grüneis, Angelos Michaelides

**Affiliations:** † Yusuf Hamied Department of Chemistry, University of Cambridge, Lensfield Road, CB2 1EW Cambridge, U.K.; ‡ Dipartimento di Fisica Ettore Pancini, Università di Napoli Federico II, Monte S. Angelo, I-80126 Napoli, Italy; § Department of Physical Chemistry and Materials Science, Faculty of Chemical Technology and Biotechnology, Budapest University of Technology and Economics, Müegyetem rkp. 3, H-1111 Budapest, Hungary; ∥ HUN-REN-BME Quantum Chemistry Research Group, Müegyetem rkp. 3, H-1111 Budapest, Hungary; ⊥ MTA-BME Lendület Quantum Chemistry Research Group, Müegyetem rkp. 3, H-1111 Budapest, Hungary; # Institute for Theoretical Physics, TU Wien, Wiedner Hauptstraße 8-10/136, 1040 Vienna, Austria; ¶ Department of Earth Sciences, University College London, Gower Street, WC1E 6BT London, U.K.

## Abstract

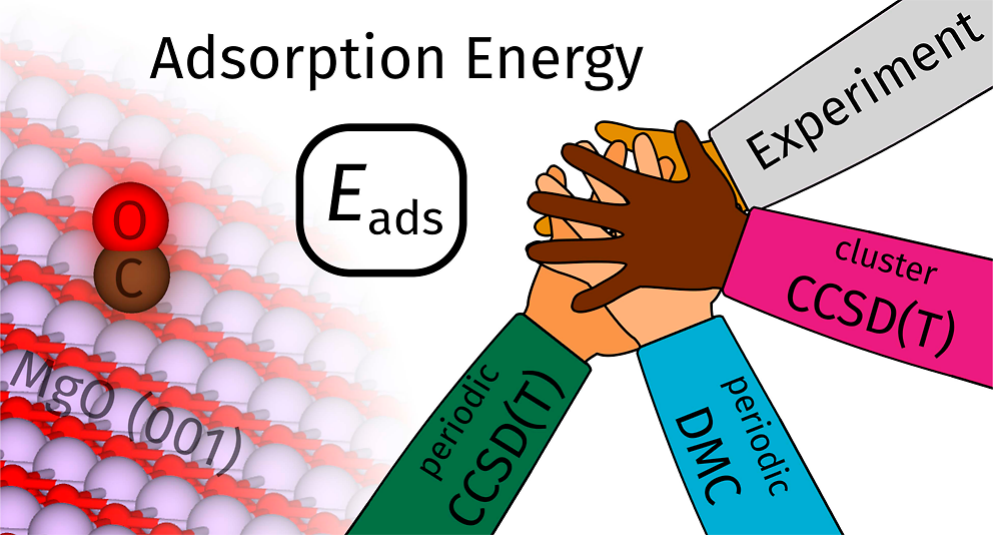

The adsorption energy of a molecule onto the surface of a material underpins a wide array of applications, spanning heterogeneous catalysis, gas storage, and many more. It is the key quantity where experimental measurements and theoretical calculations meet, with agreement being necessary for reliable predictions of chemical reaction rates and mechanisms. The prototypical molecule–surface system is CO adsorbed on MgO, but despite intense scrutiny from theory and experiment, there is still no consensus on its adsorption energy. In particular, the large cost of accurate many-body methods makes reaching converged theoretical estimates difficult, generating a wide range of values. In this work, we address this challenge, leveraging the latest advances in diffusion Monte Carlo (DMC) and coupled cluster with single, double, and perturbative triple excitations [CCSD(T)] to obtain accurate predictions for CO on MgO. These reliable theoretical estimates allow us to evaluate the inconsistencies in published temperature-programed desorption experiments, revealing that they arise from variations in employed pre-exponential factors. Utilizing this insight, we derive new experimental estimates of the (electronic) adsorption energy with a (more) precise pre-exponential factor. As a culmination of all of this effort, we are able to reach a consensus between multiple theoretical calculations and multiple experiments for the first time. In addition, we show that our recently developed cluster-based CCSD(T) approach provides a low-cost route toward achieving accurate adsorption energies. This sets the stage for affordable and reliable theoretical predictions of chemical reactions on surfaces to guide the realization of new catalysts and gas storage materials.

## Introduction

The adsorption energy (*E*_ads_) of a molecule on the surface of a material is a quantity of fundamental importance. For example, adsorption (or desorption) forms the primary rate-limiting step of many critical reactions in heterogeneous catalysis,^1,2^ with overall reaction rates determined by their *E*_ads_.^3,4^ It is also used to determine the selectivity of a surface for binding a particular molecule, relevant for the storage and sequestration of gases pertinent to energy applications.^5^ These properties depend sensitively on the value of *E*_ads_, making it vitally important to obtain this quantity accurately with either theoretical calculations or experimental measurements.

Touted as the ‘hydrogen molecule of surface science’,^6^ the CO adsorption energy onto the MgO (001) surface has served as the quintessential test for both theory and experiment.^7−14^ It is highly representative of many important processes (e.g., CO oxidation^15^ and N_2_ reduction^16^ in surface catalysis, as well as CO_2_^17^ adsorption in gas storage), and the weak van der Waals (vdW) dispersion interactions that govern the *E*_ads_ make it a stringent test. As such, a method incapable of obtaining the *E*_ads_ of CO on MgO accurately cannot be trusted to reliably predict molecule–surface interactions for more complex surface phenomena. In this context, an *E*_ads_ prediction is typically considered reliable if it reaches “chemical accuracy” of 43 meV (1 kcal/mol).^18^ This level of precision on *E*_ads_ (together with smaller temperature contributions) is essential for the dependable estimation of crucial thermodynamic properties, including chemical reaction rates.^19^

**Figure 1:**
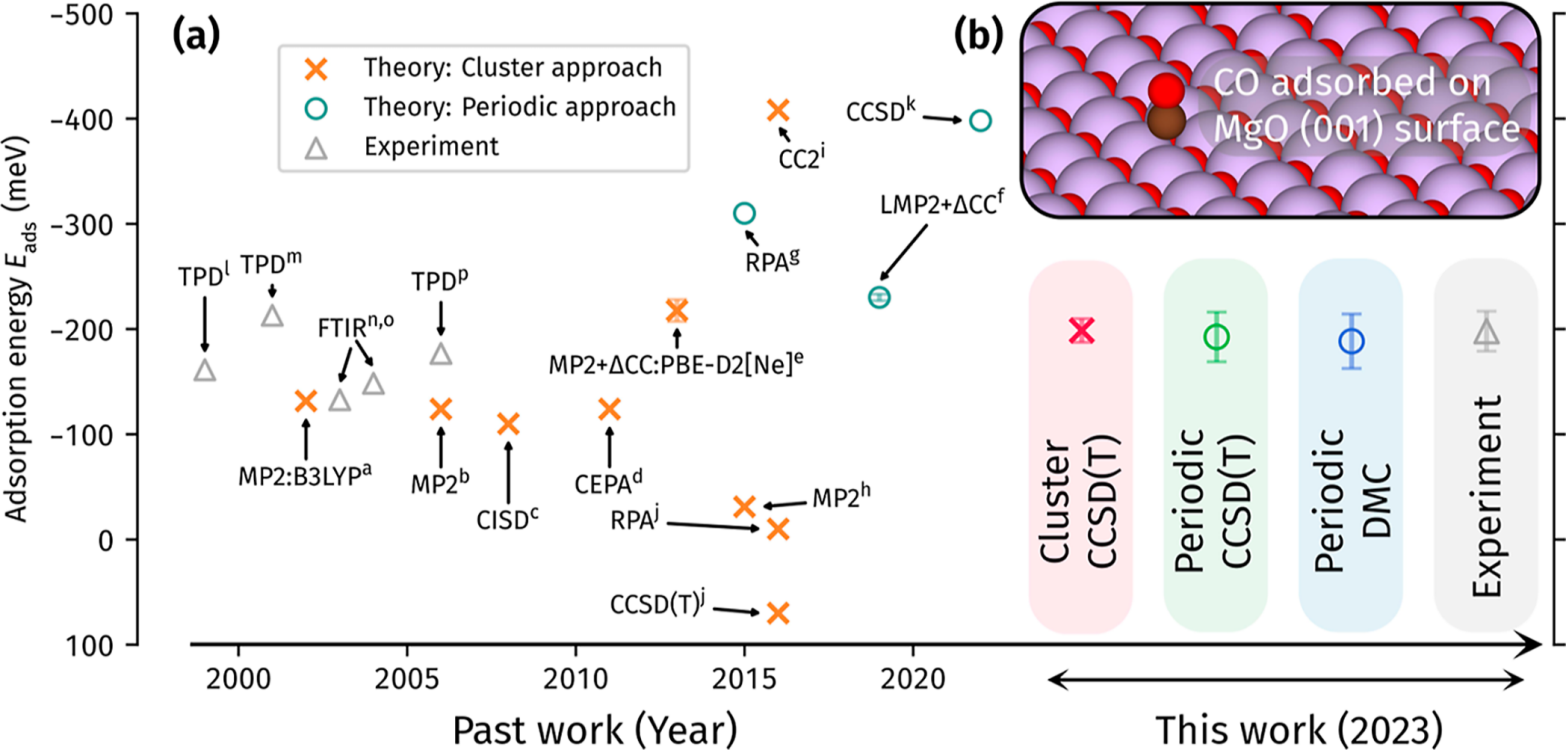
(a) Adsorption energy *E*_ads_ of CO on MgO from previous experimental and theoretical investigations. For the past theory work, we focus on many-body wave function studies employing either a cluster or periodic approach. The past experimental work involves either the Fourier transform infrared (FTIR) or the TPD technique, which we discuss in Section S9 of the Supporting Information. The CO on MgO system is visualized in the top panel of (b), and in its bottom panel, we give accurate estimates to *E*_ads_ from this work utilizing CCSD(T) with a cluster approach, CCSD(T) with a periodic approach, and DMC with a periodic approach. A best estimate of the experimental value has also been made by reanalyzing the previous experimental work with an improved pre-exponential factor (discussed in the text). Error bars have been determined for all estimates made from this work. References for past simulation work are as follows: (a) Ugliengo and Damin.^20^ (b) Herschend et al.^21^ (c) Qin et al.^22^ (d) Staemmler.^23^ (e) Boese and Sauer.^24^ (f) Alessio et al.^25^ (g) Bajdich et al.^26^ (h) Li et al.^27^ (i) Heuser and Höfener.^28^ (j) Mazheika and Levchenko.^29^ (k) Mitra et al.^30^ References for past experiments are as follows: (l) Wichtendahl et al.^31^ (m) Dohnálek et al.^32^ (n) Spoto et al.^33^ (o) Spoto et al.^34^ (p) Sterrer et al.^35^

Unfortunately, obtaining an accurate *E*_ads_ is highly challenging for both theory and experiment. Despite a large body of experimental and theoretical investigations (Figure 1a), the *E*_ads_ of CO on MgO is still under debate. Even nominally accurate many-body theoretical methods (Figure 1a) can produce a range of nearly 500 meV (11 kcal/mol) on *E*_ads_, encompassing predictions going from weak physisorption to moderate chemisorption. At room temperature, this range can lead to over 8 orders of magnitude change in reaction rate predictions. Experimental measurements have covered a similar range in the past,^11−14^ while recent estimates (Figure 1a) have settled to between −133 and −208 meV, this range is still too large. Crucially, it has not been possible to establish agreement on the CO on MgO *E*_ads_ between multiple theoretical approaches and multiple experiments at the same time (see Section S1.2 of the Supporting Information).

Modeling the weak vdW interactions that govern the binding of CO on MgO requires a rigorous treatment of its electronic structure. This raises questions over common electronic structure methods, such as density functional theory (DFT) or second-order Møller–Plesset perturbation theory (MP2). The former does not naturally incorporate vdW dispersion in its standard approximations (although approaches^36−38^ are available), while the latter lacks higher-order dispersion effects.^39^ For modeling these interactions in small molecules, the methods of choice are quantum diffusion Monte Carlo (DMC)^40^ and coupled cluster with single, double, and perturbative triple particle-hole excitation operators [CCSD(T)].^41^ While both DMC^42−46^ and CCSD(T)^47−51^ have been successfully used for several surface adsorption (and even dissociation/reaction^52,53^) problems, there remain open questions on their reliability for extended systems (i.e., surfaces and large molecules). For example, recent work^54^ has indicated significant differences in the interaction energies between large and complex molecules (many analogous to molecule–surface interactions). These unresolved questions prompt a fresh review of the CO on MgO system to clarify the origin of its discrepancies among theoretical techniques.

Applying DMC or CCSD(T) to surface problems is highly challenging because of the steep scaling of their computational complexity with the number of atoms. With these methods, surfaces can be modeled either as a finite cluster or a repeating supercell slab, termed cluster and periodic approaches, respectively. To date, neither DMC nor CCSD(T) has been applied to examine CO adsorption on MgO with a periodic approach. While CCSD(T) with a cluster approach, termed cluster CCSD(T) hereafter, has been previously performed, it is difficult to converge. For example, the aforementioned 500 meV range arises from cluster CC-based *E*_ads_ estimates that are not adequately converged. Here, the challenge lies in simultaneously converging both the surface model (size) and the electronic structure settings. The former requires large system sizes (both cluster and periodic) to reach the bulk (infinite size) limit and a dilute CO coverage, while the latter requires large basis sets and the inclusion of correlation from electrons in subvalence metal shells. These requirements all contribute to a significant computational burden that can become intractable.

In this work, we reach a consensus for the CO on MgO *E*_ads_, achieving agreement between theory and experiment. For theory, we leverage the latest advances in periodic DMC, periodic CCSD(T), and cluster CCSD(T) to produce three high-quality estimates of the *E*_ads_. With this, we establish an agreement between all three theoretical techniques to subchemical accuracy. This has allowed us to evaluate and understand the inconsistencies in previous theoretical calculations and experimental measurements. For example, we establish that the discrepancies among previous temperature-programed desorption (TPD) experiments arise predominantly from the use of different pre-exponential factors. Subsequently, we derive new *E*_ads_ values for these TPD experiments with a more accurate pre-exponential factor (while removing thermal and zero-point contributions). This effort has made it possible for this study to become the first to establish a consensus between a variety of theoretical techniques and multiple experimental measurements. These estimates from both theory and experiment place the CO on MgO system squarely in the physisorption regime, all lying within the −199 ± 11 meV range set by our best *E*_ads_ estimate from the cluster CCSD(T) technique. Crucially, we demonstrate that our employed cluster CCSD(T) technique, combining the recently developed SKZCAM protocol^55^ with reduced-scaling CCSD(T), can achieve its high accuracy at a low cost comparable to (hybrid) DFT. This opens the door for its use as a routine benchmark tool^56−58^ as well as within high-throughput frameworks for predicting new and improved catalyst^59^ and gas storage materials.^60^

## Methods

Before assessing the final *E*_ads_ obtained for the three theoretical techniques [cluster CCSD(T), periodic CCSD(T), and periodic DMC], we will discuss how we have been able to reach such high-quality estimates in this section. Each theoretical technique approaches the final *E*_ads_ differently based on the choice of the electronic structure method [CCSD(T) or DMC] and surface model (periodic or cluster). For example, CCSD(T)^61^ tackles the many-electron Schrödinger equation via an expansion of electronic configurations (using particle-hole excitation operators) from a reference wave function, while DMC^62^ achieves this via an imaginary time projection to the ground state from a trial wave function. Accordingly, these two electronic structure methods depend on different factors, such as the basis-set size for CCSD(T) and the time step for DMC as described in Section S5 of the Supporting Information. In fact, to reach sufficient accuracy, this even affects how we go about computing the *E*_ads_, which we discuss first below. Thereafter, we will also describe how the separate surface models reach the bulk limit and dilute coverage regimes.

### Computing Adsorption Energy

The primary quantity of interest in this work is the adsorption energy, which physically represents the energy released when a CO molecule in the gas-phase adsorbs onto a pristine MgO surface and can be defined as
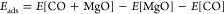
1where *E*[CO + MgO], *E*[MgO], and *E*[CO] are the energies of the CO on MgO (CO + MgO), pristine MgO, and gas-phase CO systems, respectively. In practice, we actually compute the interaction energy, where we have two definitions depending on the theoretical technique
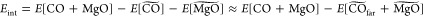
2

The first definition is similar to *E*_ads_ but calculates the energy of the separate  and  systems with structures frozen from the CO + MgO system (as indicated by  and , respectively). Computing *E*_int_ (over *E*_ads_ directly) allows for basis-set superposition error (BSSE) corrections^63^ to be applied to cluster CCSD(T) calculations. For periodic DMC and periodic CCSD(T), we use the second definition of *E*_int_, where the  system corresponds to the frozen  displaced >5 Å away from the frozen surface, both taken from the CO + MgO system. It differs from the (formal) first definition of *E*_int_ by less than 5 meV (Section S5.2 of the Supporting Information) and was used to mitigate finite-size errors^44^ for both calculations, while also enabling larger timesteps to make DMC more economical.

Reaching the final *E*_ads_ from *E*_int_ then requires the addition of a Δ_geom_ term; it represents the energy required to relax the separate frozen CO and MgO geometries back into their equilibrium geometries. As obtaining forces (and thus equilibrium geometries) is challenging for both CCSD(T) and DMC, the CO, MgO, and CO + MgO structures as well as Δ_geom_, a small term, were approximated at the DFT level. Specifically, we chose the revPBE-D4 exchange–correlation functional^64^ (and dispersion treatment^65^) due to its reasonable *E*_ads_ and geometrical parameters compared to CCSD(T) and experiment (see Section S3 of the Supporting Information). As discussed in Section S4 of the Supporting Information, the errors arising from the use of revPBE-D4 geometries have been conservatively estimated by assessing its effect on an ensemble of high-quality DFT functionals along Jacob’s ladder.^36,66^

### Periodic Approaches

**Figure 2:**
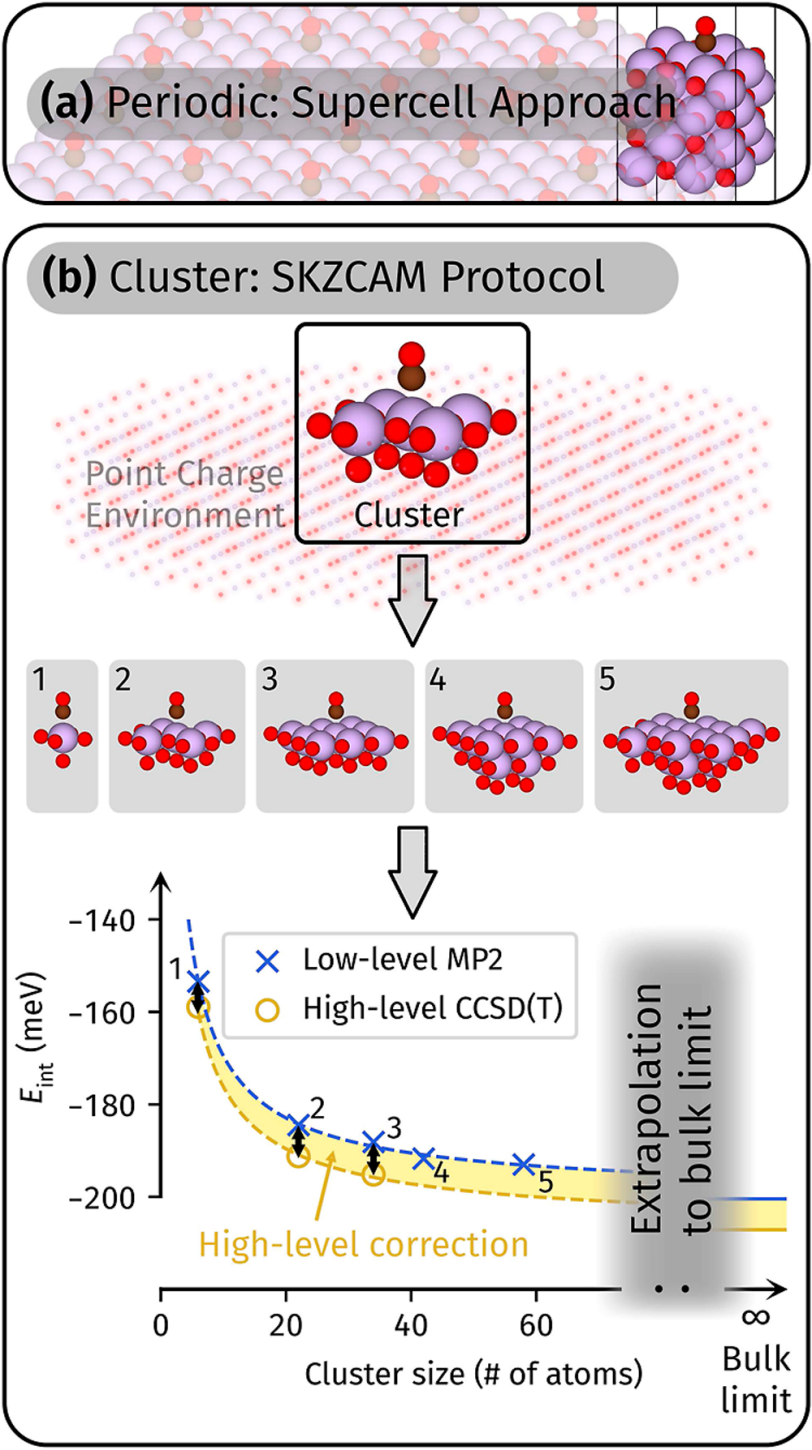
Schematic of the (a) periodic supercell approach and (b) cluster approach used in this study to compute the adsorption energy of CO on MgO. We performed both DMC and CCSD(T) with the periodic supercell approach. The cluster approach in (b) is based on our recently developed SKZCAM protocol,^55^ which generates a series of quantum clusters of increasing size under an electrostatic embedding framework (see top panel). We have computed the interaction energy *E*_int_ for the first three (five) clusters at the LNO-CCSD(T) (MP2) level, which are numbered in the bottom panel of (b). The MP2 bulk limit was estimated by extrapolating this series of clusters. A subsequent high-level correction to the CCSD(T) level was estimated from a subset of these clusters via the LNO-CCSD(T) approach.

Assuming converged electronic structure methods (Section S5 of the Supporting Information), we must ensure that the surface models used (see Figure 2) have converged to the bulk limit and dilute CO coverage regimes. Periodic approaches can achieve this in a straightforward fashion via the supercell approach (Figure 2a) by increasing the surface supercell size and number of slab layers. As shown in Section S5.4 of the Supporting Information, we find that a four-layer (4L) (4 × 4) supercell of the MgO (001) surface is sufficient to converge *E*_ads_ to less than 1 meV at the DFT level. We performed periodic CCSD(T) with the Cc4s code^50,67−69^ and periodic DMC with CASINO.^70^ Even with the latest advances, direct calculation (at converged settings) on the 4L (4 × 4) supercell can be computationally expensive for both CCSD(T) and DMC, although the more favorable system size scaling of DMC can enable such systems to be tackled.^52^ Instead, we have computed *E*_int_ on a 2L supercell cleaved from the original 4L supercell and, in the vein of Pople’s model chemistry,^71^ approximated the remaining (much) smaller contributions with computationally economical methods, as elaborated in Section S6 of the Supporting Information.

### Cluster Approaches

Cluster approaches work by placing a finite cluster within appropriate embedding environments. They naturally provide dilute coverage estimates, but convergence toward the bulk limit is challenging. As discussed in our previous study,^55^ the convergence of a finite cluster depends on interdependent factors such as (1) embedding approach (e.g., mechanical;^6^ electrostatic,^72^ incremental^73^ or quantum^74−78^); (2) shape; (3) size; and (4) charge of the cluster. The use of steep scaling methods such as CCSD(T) severely limits the cluster size that can be reached. In this study, we use the local natural orbital (LNO) scheme^79−82^ [LNO-CCSD(T)] in Mrcc(83) to further extend the feasible system sizes while maintaining high accuracy (Section S7 of the Supporting Information). The challenge is then to keep the (quantum) cluster small enough to make well-converged LNO-CCSD(T) computations routinely affordable while also reaching the bulk limit.

Our recently proposed SKZCAM protocol is particularly suited for tackling this challenge. It is based upon the electrostatic embedding approach^72,84−86^ (top panel of Figure 2b) and provides the design rubrics to generate a series of quantum clusters of systematically increasing size (middle panel of Figure 2b). We have shown previously^55^ and here (bottom panel of Figure 2b) that these clusters converge smoothly and rapidly to the bulk limit. Although initially devised for calculating oxygen vacancy formation energies, it has been extended to encompass adsorption on metal-oxide surfaces as part of this study. We take advantage of the smooth convergence with cluster size in the SKZCAM protocol to extrapolate (see Section S7.1) a small number of clusters to the bulk limit. This extrapolation (inspired by the form of the Jost correction^87^ and empirical dispersion corrections^37^) is expected to naturally incorporate any missing long-range polarization and dispersion effects. As shown in the bottom panel of Figure 2b and Table S10 of the Supporting Information, only the first five clusters are required to converge to within 5 meV.

While the largest cluster size (∼60 atoms) is amenable at the MP2 level, it is intractable with canonical CCSD(T). Fortunately, convergence to the bulk limit of CCSD(T) can be accelerated by evaluating a LNO-CCSD(T) level correction to the bulk limit MP2 for a series of smaller clusters in the fashion of the ΔCC correction from Boese et al.^24^ This correction is highly accurate because another quality of the SKZCAM protocol is the good cancellation of finite-size errors between many-body methods such as MP2 and CCSD(T) across its clusters. Specifically, we find deviations of only 3 meV in this correction across the first three clusters of the SKZCAM protocol. Note that this correction is different for every new molecule–surface system. The resulting computations in this protocol require only a few days on a single computer node, easily accessible in commodity computer clusters.

## Results

### Agreement between Many-Body Methods

As discussed in the Methods, the final *E*_ads_ we obtain for each of the three techniques is actually composed of several terms, where besides Δ_geom_, *E*_int_ itself consists of several contributions. As shown in Section S6 of the Supporting Information, each of these terms has been carefully converged, with conservative error bars estimated for the most important terms. With this effort, we come to a final *E*_ads_ estimate (in meV) of −199 ± 11 for cluster CCSD(T), −193 ± 24 for periodic CCSD(T), and −188 ± 26 for periodic DMC (summarized in Table 1). This agreement is better than chemical accuracy; in fact, we reach subchemical accuracy with a maximum deviation of 11 meV (1 kJ/mol) across the three theoretical techniques, smaller than their error bars. These *E*_ads_ values place the adsorption behavior of CO on MgO squarely in the physisorption regime, right in the middle of the aforementioned large 500 meV range across previous theoretical calculations (Figure 1). To give some perspective, the H_2_O monomer, known to chemisorb on MgO, has an *E*_ads_ in the −480 to –550 meV^25,42^ range, close to some previous theoretical estimates for CO on MgO.

**Table 1:** Comparison of the Final *E*_ads_ Estimates between the Cluster CCSD(T), Periodic CCSD(T), and Periodic DMC Techniques as Well as Our Best Estimate from Experiment^a^

technique	*E*_ads_ (meV)	cost (kCPUh)	max RAM (GB)
cluster CCSD(T)	**–199** ± **11**	∼20	∼20
periodic CCSD(T)	**–193** ± **24**	∼200	∼3000
periodic DMC	**–188** ± **26**	∼1000	negligible
experiment	**–198** ± **19**	N/A	N/A

a Estimates on the computational cost in 1000 CPU core hours (kCPUh) and maximum RAM usage in gigabytes (GB) are also given. No RAM usage has been given for DMC because it uses a negligible amount relative to CCSD(T).

Reaching agreement for the CO on MgO *E*_ads_ across fundamentally distinct electronic structure methods [DMC and CCSD(T)] and surface models (cluster and periodic) that have been systematically converged gives us confidence in using these estimates to evaluate past theoretical and experimental literature. In particular, the low cost of the cluster CCSD(T) approach (elaborated in the Discussion) allows for effects of electronic structure settings, such as basis-set size, frozen core size, and cluster size, on the *E*_ads_ to be studied. For example, in Section S8 of the Supporting Information, we show that inadequate basis-set size, large frozen core size (i.e., only including valence electrons in the many-body correlation treatment), and small cluster size all lead to weaker binding (i.e., less negative *E*_ads_). On the basis of this convergence analysis, we have been able to attribute many of the underestimated literature values to inadequate convergence of these properties. Similarly, we show that the studies that overestimate the binding strength largely result as they do not correct for BSSE, which becomes particularly strong for small basis sets. The advances in accuracy of the techniques in this study point toward an agreement with only the work from Sauer’s group, first computed by Boese and Sauer^24^ and then by Alessio et al.,^25^ reaffirming the reliability of their High-level:Low-level approach.^24,25,88−91^

### Re-evaluating Previous Experimental Measurements

Our reliable theoretical estimates now give us the opportunity to evaluate the discrepancies between past experiments. These previous experiments, of which there are many, have spanned a broad 300 meV range (see Section S2 of the Supporting Information). As discussed before^6,9,92^ and in Section S9 of the Supporting Information, some of these measurements are not reliable, and we focus only on the recent (three) TPD experiments. In their original TPD measurements, (Arrhenius) activation energies (*E*_act_) of −140, −192, and −155 meV were measured by Wichtendahl et al.,^31^ Dohnálek et al.,^32^ and Sterrer et al.,^35^ respectively. Notably, there is still a deviation^24^ of 52 meV (>1 kcal/mol) that is too large.

**Figure 3:**
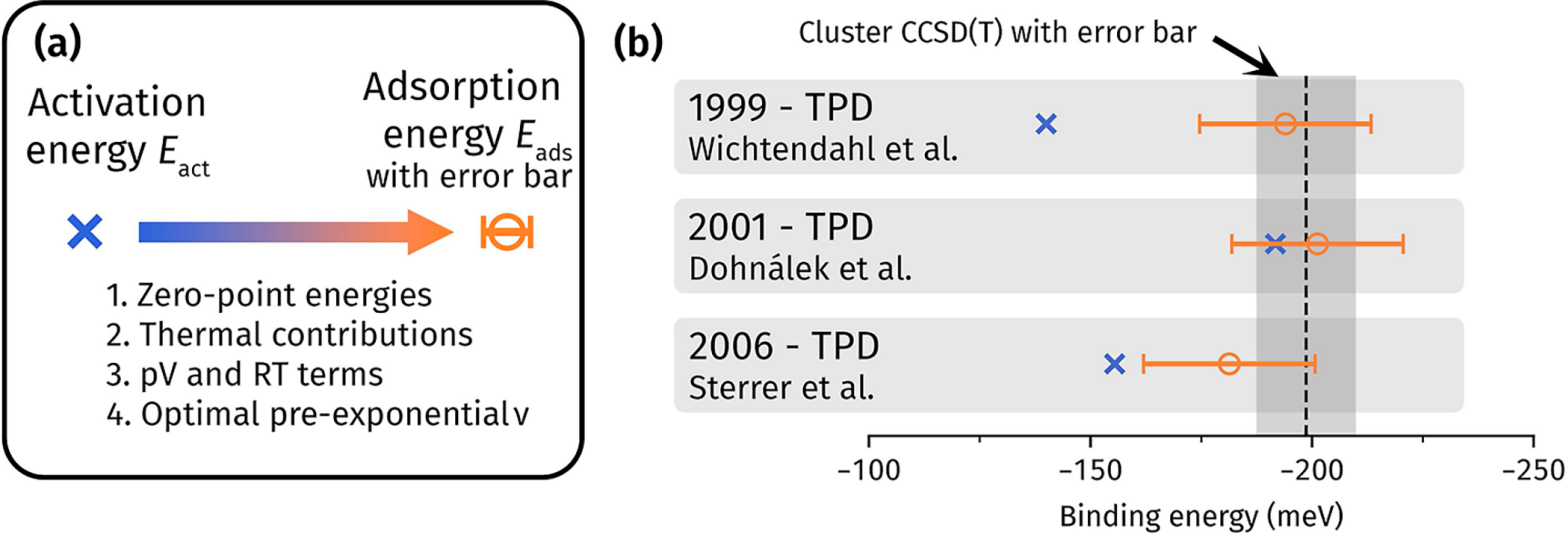
(a) Effects considered during conversion of the Arrhenius activation energies *E*_act_ in TPD experiments to adsorption energies *E*_ads_ suitable for comparison to theory. (b) Resulting *E*_ads_ converted from *E*_act_ match to within error bars with our cluster CCSD(T) estimate for all three TPD experiments (from Wichtendahl et al.,^31^ Dohnálek et al.,^32^ and Sterrer et al.^35^).

To compare these TPD experiments against our theoretical calculations, the original *E*_act_ values must be converted into *E*_ads_. The importance of this conversion has only been noted in a handful of recent CO on MgO studies.^10,24^ Typically, it involves removing thermal and zero-point contributions, as well as pV and RT terms (i.e., effects 1, 2, and 3, but not 4, in Figure 3a). It is common to compute these terms accurately using DFT (as performed in Section S9.2 of the Supporting Information). However, this only constitutes a constant shift of −19 meV for each TPD experiment and does not solve the large noted deviation.^24^ For example, with only these effects accounted for, Wichtendahl et al., Dohnálek et al., and Sterrer et al. predict *E*_ads_ of −156, −208, and −172 meV, respectively (see Section S2 of the Supporting Information and Figure 1a).

Our theoretical estimates (between −188 and −199 meV) are in the middle of the above *E*_ads_ range, with Wichtendahl et al. and Sterrer et al. underestimating while Dohnálek et al. overestimating. This differing behavior points toward the pre-exponential factor (ν) being the culprit. For example, ν is not typically known and commonly assigned to log(ν) = 13 (e.g., by Wichtendahl et al. and Sterrer et al.), while Dohnálek et al. have estimated (with large ±2 error bars) it to be log(ν) = 15. Since these original experiments, ν has received considerable attention,^38,93,94^ and, importantly, an estimate of log(ν) = 13.8 ± 1.6 has been given by Campbell and Sellers,^95^ agreeing with a theoretical estimate [log(ν) = 14.2] from Nygren and Pettersson.^9^ Thus, there is now the prospect of making corrections^38^ toward a better ν value (effect 4 in Figure 3a). In Figure 3b, we have made these ν corrections to *E*_act_, combining it with the aforementioned thermal contributions and using a newer analysis^95^ of the TPD curve from the original study by Wichtendahl et al. The resulting experimental *E*_ads_ range falls to within 20 meV (i.e., better than chemical accuracy), and all three experiments now agree with our theoretical estimates, where Wichtendahl et al., Dohnálek et al., and Sterrer et al. predict *E*_ads_ of −194, −201 and −181 meV, respectively, with ±19 meV error bars arising from uncertainty in ν.

## Discussion

The achieved agreement is a testament to the algorithmic and methodological developments made in the past decades on all three theoretical techniques to enable such high accuracy at a tractable computational cost. As discussed previously, the accuracy and reliability of the cluster CCSD(T) *E*_ads_ value have been made possible with the SKZCAM protocol combined with the recent advances in local approximations to CCSD(T) [e.g., LNO-CCSD(T),^79−82,96^ DLPNO-CCSD(T),^97−101^ PNO-LCCSD(T),^102−104^ etc.]. While canonical CCSD(T) could only be performed for the smallest quantum clusters of up to 1–2 dozen atoms,^82^ LNO-CCSD(T) can tackle molecules involving hundreds of atoms^54,81^ and ionic crystal clusters of around 100 atoms.^55^ For periodic DMC, the introduction of ccECP pseudopotentials^105,106^ gives confidence in calculations involving elements beyond the first row, while the ZSGMA^107,108^ algorithm and determinant localization approximation (DLA)^109^ enable larger timesteps for the same accuracy. While DMC has had a long history spanning several decades,^42,46,110−112^ the periodic CCSD(T) technique has only come into maturation in recent years^113−116^ and besides the significant algorithmic improvements,^69,117^ it is particularly the recent developments in finite-size corrections^50,53,67^ that have enabled chemical accuracy to be reached for CO on MgO and indeed other surface adsorption problems.^118,119^

We compare the computational costs of the three techniques in Table 1. While a one-to-one comparison cannot be made because the calculations were performed on different computing architectures, it is clear that cluster CCSD(T) is cheaper by 1 or 2 orders of magnitude compared to either of the periodic techniques. In fact, this cost is comparable to periodic hybrid DFT calculations, which takes ∼1k CPU-hours to compute. From previous work, we have found that the (cluster-based) SKZCAM protocol, combined with the reduced scaling and efficient implementation of LNO-CCSD(T), can actually become cheaper than periodic hybrid DFT for more complex surfaces such as TiO_2_. The cluster CCSD(T) calculations require a small amount of memory (∼20 GB on a single node), amenable on standard computing hardware (typically containing >128 GB on a single node). On the other hand, periodic CCSD(T) can require ∼3000 GB of RAM distributed across high-memory nodes. It should be noted that while periodic DMC  has been more expensive than periodic CCSD(T)  for the CO on MgO system studied here, its better scaling with system size, excellent parallelization (across computer nodes), and low memory requirements should enable it to be more efficient for larger surfaces and molecules.

The true *E*_ads_ value for each technique (i.e., when both electronic structure settings and the surface model are converged) is anticipated to lie within its respective error range in Table 1. Out of the three theoretical techniques, the cluster CCSD(T) calculation has the lowest error bars, and this is achieved by design, thanks to the SKZCAM protocol. For example, finite-size errors from the MP2 extrapolation to the bulk limit can be estimated by including more clusters into the formula and likewise the high-level correction up to CCSD(T), as discussed in Section S7 of the Supporting Information. For this reason, we consider the *E*_ads_ estimate of −199 ± 11 meV by the cluster CCSD(T) technique to be the best estimate. We chose not to combine all three theoretical techniques into one best estimate because their errors have distinct origins and behaviors. For example, the periodic CCSD(T) error bars are systematic, arising from an incomplete basis set, while the errors are stochastic for periodic DMC.

It will form the topic of future work whether the high accuracy and low cost of this cluster approach will persist for other molecule–surface systems. Tackling surfaces with metallic^120,121^ or covalent^122^ character will require the use of alternative embedding approaches. In particular, for (transition) metals, new effective core potentials^123^ and developments in applying CC-based theories to metals^124,125^ should now enable high-accuracy and low-cost cluster approaches to be developed for these systems. We expect that the SKZCAM protocol’s core principle—extrapolating bulk properties from a small series of well-constructed clusters—will persist for these covalent^122^ and metallic^121^ systems as well. Future periodic CCSD(T) calculations of metal surfaces will also become more economical and feasible. Thanks to recent developments,^117^ the previous requirement for numerous twist averages in metals, to address independent-particle finite-size errors, has been streamlined to a single special twist angle, promising a substantial reduction in costs by 1 or 2 orders of magnitude.

The accuracy of the three theoretical techniques has come to such high precision that it is now possible to benchmark the accuracy of experiments. In particular, it has demonstrated the necessity of utilizing accurate pre-exponential factors in TPD experiments to reach reliable agreement. This means that while agreement has been achieved previously for theoretical calculations and specific experiments, these must be viewed with skepticism. Out of the re-evaluated *E*_ads_ values in Figure 3b, we expect the reanalyzed *E*_ads_ estimates from the TPD experiments by Wichtendahl et al. and Dohnálek et al. to provide a more accurate estimate than Sterrer et al. as they involve lower CO surface coverages (see Section S9.3 of the Supporting Information). As such, we take the average of the two to come to the best experimental estimate of –198 ± 19 meV, which we use in Figure 1b and Table 1. Our cluster CCSD(T) estimate of −199 ± 11 meV demonstrates near-exact agreement to this experimental estimate, and its smaller error bars underscore its status as the best estimate of the CO on MgO *E*_ads_ out of all theoretical calculations and experimental measurements.

## Conclusions

In summary, we have resolved the value of the adsorption energy (*E*_ads_) for CO on MgO to −199 ± 11 meV, achieving consensus between three independent theoretical calculations [cluster CCSD(T), periodic CCSD(T), and periodic DMC] and three separate TPD experiments. It establishes both DMC and CCSD(T) as methods that have matured sufficiently to benchmark surface phenomena. For example, we used reliable theoretical estimates to assess and understand the discrepancies in the previous literature (both theory and experiment). In particular, we demonstrate that the differences between previous experimental TPD measurements and our theoretical estimates arise from differing pre-exponential factors. A subsequent re-evaluation with a more precise pre-exponential factor has now allowed for the agreement to be achieved, highlighting the importance of considering this factor in future work. Furthermore, we show that the cluster CCSD(T) technique, made possible with the SKZCAM protocol and the reduced scaling LNO-CCSD(T) method, demonstrates high accuracy at low cost; requiring only a few days on a single computer node.

While agreement between theory and experiment has been achieved before for specific surfaces,^6,49^ the SKZCAM protocol^55^ used here promises the ability to generalize this accuracy to other surfaces and properties systematically, amenable for automated high-throughput calculations. Combined with its accuracy and low cost, these properties of the SKZCAM protocol open the door toward studying the interaction of many molecules and surfaces simultaneously at reference quality with the cluster CCSD(T) technique. With this, we can create large benchmark databases suitable for assessing the quality of DFT functionals—currently sorely lacking for metal-oxide surfaces.^93^ Furthermore, we can now go beyond adsorption to study catalytic reaction steps on technologically relevant surfaces. Here, the combination of theoretical calculations and experimental measurements, now capable of reaching a consensus, will enable the unveiling of precise mechanistic insights^126,127^ into these surface reaction phenomena.

## Supporting Information Available

The input and output files associated with this study and all analysis can be found on GitHub at github.com/benshi97/Data_CO_on_MgO or viewed (and analyzed) online on Colab. The Supporting Information is available free of charge at https://pubs.acs.org/doi/10.1021/jacs.3c09616.

Discussion of past CO on MgO estimates, estimating the uncertainty of the revPBE-D4 geometry, details on computational techniques and convergence, final E_ads_ estimates and computational costs, ,and re-evaluation of previous experimental E_ads_ estimates (PDF)
