# Supplementary material for: Many-Body Methods for Surface Chemistry Come of Age: Achieving Consensus with Experiments
Source: J Am Chem Soc. 2023 Nov 10;145(46):25372–81. doi: 10.1021/jacs.3c09616 (PMC10683001; doi:10.1021/jacs.3c09616)
Supplement: Supplementary file 1 — ja3c09616_si_001.pdf [file ja3c09616_si_001.pdf]

Supporting Information: Many-body methods for surface chemistry come of age: Achieving consensus with experiments

Benjamin X. Shi,[†] Andrea Zen,[‡,#] Venkat Kapil,[†] Péter R. Nagy,[¶,§,‖] Andreas Grüneis,[⊥] and Angelos Michaelides[*,†]

†*Yusuf Hamied Department of Chemistry, University of Cambridge, Lensfield Road, Cambridge CB2 1EW, United Kingdom*

‡*Dipartimento di Fisica Ettore Pancini, Università di Napoli Federico II, Monte S. Angelo, I-80126 Napoli, Italy*

¶*Department of Physical Chemistry and Materials Science, Faculty of Chemical Technology and Biotechnology, Budapest University of Technology and Economics, Műegyetem rkp. 3., H-1111 Budapest, Hungary*

§*ELKH-BME Quantum Chemistry Research Group, Műegyetem rkp. 3., H-1111 Budapest, Hungary*

‖*MTA-BME Lendület Quantum Chemistry Research Group, Műegyetem rkp. 3., H-1111 Budapest, Hungary*

⊥*Institute for Theoretical Physics, TU Wien, Wiedner Hauptstraße 8-10/136, 1040 Vienna, Austria*

#*Department of Earth Sciences, University College London, Gower Street, London WC1E 6BT, United Kingdom*

E-mail: am452@cam.ac.uk

Contents

S1 Discussion of past CO on MgO literature **S-3**

S1.1 Theory S-3

S1.2 Experiment S-4

S2 CO on MgO E_{ads} estimates from the literature **S-5**

S3 Benchmarking the revPBE-D4 geometry **S-6**

S4 Uncertainty in geometrical relaxation (Δ_{geom}) **S-7**

S5 Computational details and convergence **S-8**

S5.1 Cluster CCSD(T) S-8

S5.2 Periodic CCSD(T) S-9

S5.3 Periodic DMC S-12

S5.4 DFT S-13

S6 Details on E_{ads} estimates and computational costs **S-14**

S7 SKZCAM protocol and its convergence **S-16**

S7.1 Extrapolation to MP2 bulk limit S-16

S7.2 ΔCC correction S-18

S8 Analysis of prior CO on MgO simulations **S-19**

S9 Reaching accurate experimental E_{ads} **S-21**

S9.1 Converting TPD E_{act} to H_{ads} S-23

S9.2 Converting H_{ads} to E_{ads} S-23

S9.3 Comparing final experimental estimates S-24

References **S-26**

We provide additional supporting data as well as contextual information to the main text here. All output files are provided on GitHub, which contains a Jupyter Notebook file that analyzes the data. This data can also be viewed and analyzed on the browser with Colab.

S1 Discussion of past CO on MgO literature

S1.1 Theory

Most early work applying many-body methods (Fig. 1a of the main text) to the CO on MgO system have employed cluster approaches as it requires smaller system sizes than periodic approaches. Cluster approaches work by placing a finite-cluster within appropriate embedding environments, of which many flavors are possible. For example, Ugliengo *et al.*[S1] embedded the finite clusters mechanically into DFT via a hybrid high-level:low-level quantum mechanical (HL:LL) method (in the fashion of Morokuma's ONIOM[S2]), where second-order Møller Plesset perturbation theory (MP2) was chosen as the high-level. On the other hand, Herschend *et al.*[S3] and Qin *et al.*[S4] embedded the cluster electrostatically into a field of point charges and Staemmler[S5] performed a many-body expansion of the correlation energy via the method of increments. These early works were confined to methods with well-known deficiencies, such as the lack of higher-order dispersion effects in MP2. As a result, reliable estimates of E_{ads} cannot be guaranteed even when the electronic structure quality and models are converged – already a challenging task in itself.

The high-level method of choice is coupled cluster (CC) with single, double and perturbative triple particle-hole excitation operators [CCSD(T)]. It is considered the 'gold-standard' of quantum chemistry and is capable of reaching sub-chemical accuracy within its domain of applicability. The first study to incorporate CCSD(T) for CO on MgO adsorption was by Boese *et al.*,[S6] where it formed a correction (performed on a small cluster) on top of MP2 in the HL:LL approach. This study came to a surprising E_{ads} value of -220 meV, different from earlier experimental and simulation estimates. It was reproduced again in a later study from

the same group by Alessio *et al.*,[S7] which also incorporated periodic local MP2[S8] calculations to validate the cluster-based approach. Unfortunately, no other studies[S9–S13] since have been able to produce such a value. In fact, the newer works involving more sophisticated CC techniques cover a range of almost 500 meV (12 kcal/mol), with one end being a periodic CCSD calculation in 2022 by Mitra *et al.*[S13] and the other end being a cluster CCSD(T) calculation in 2016 by Mazheika and Levchenko.[S11]

S1.2 Experiment

While experimental estimates to the CO on MgO E_{ads} have frequently been used to assess the quality of past theoretical work, the large discrepancies between experiments over the years can make reliable comparisons challenging. These past experimental estimates have ranged from weakly physisorbed[S14,S15] (~ -130 meV) to moderately chemisorbed (~ -400 meV).[S14,S15] While the variations in recent estimates (Fig. 1a) have largely settled to between -140 and -240 meV, this range is too large to enable reliable benchmarks. For example, the E_{ads} of the CO on MgO system was considered largely resolved to around -130 meV between 2003 and 2012, with simulations at the time (Fig. 1a) agreeing with estimates by Wichtendahl *et al.*[S16] (alongside several other studies). The agreement was actually fortuitous because the E_{ads} computed from theory was being incorrectly compared to the adsorption enthalpy H_{ads} measured from experiments. As discussed in Sec. S2, removing temperature and zero-point effects contribute a -19 meV shift to convert H_{ads} to E_{ads}, making the agreement much worse (see Fig. 1a of the main text). On the other hand, later work in 2013 by Boese *et al.*[S6] with the MP2+ΔCC:PBE-D2 approach found excellent agreement to a separate TPD estimate by Dohnálek *et al.*[S17] in 2001, while agreement was unsatisfactory against Wichtendahl *et al.*.

S2 CO on MgO E_{ads} estimates from the literature

The adsorption energy (E_{ads}) and adsorption enthalpy (H_{ads}) for both simulation and experimental work from the past years are presented in Table S1. Experiments provide either H_{ads} or activation energies E_{act} (for the case of TPD). We have converted these quantities from experiment into an E_{ads} with the formula: $H_{\mathrm{ads}} = E_{\mathrm{ads}} + E_{\mathrm{cor}}$, where E_{cor} is estimated to be 19 meV (see Sec. S9). These experimental estimates are plotted in Fig. 1a of the main text. It should be distinguished that in Sec. S9.1, we will also include an additional correction for the pre-exponential factor on top of the E_{ads} currently given in Table S1. For the E_{ads} from simulations, we also performed the (opposite) conversion to H_{ads}.

Table S1: The adsorption energy E_{ads} and adsorption enthalpy H_{ads} (the activation energy E_{act} is given instead for TPD) of previous computational and experimental work in meV. Additional details (including method and publication year) are given for each study.

Reference	E_{ads}	$H_{\mathrm{ads}}/E_{\mathrm{act}}$	Method	Year	Details
			**Experiment**		
Furuyama *et al.*[S14]	-184	-165	Isothermal adsorption	1978	
Paukshtis *et al.*[S15]	-174	-155	IR spectroscopy	1981	
Henry *et al.*[S18]	-439	-420	Auger spectroscopy	1991	
He *et al.*[S19]	-448	-429	Isothermal adsorption	1992	
Wichtendahl *et al.*[S16]	-156	-140	TPD	1999	pre-exponential $\log(\nu) = 13$
Dohnálek *et al.*[S6,S17]	-208	-192	TPD	2001	$\log(\nu) = 15 \pm 2$
Spoto *et al.*[S20]	-133	-114	FTIR spectroscopy	2003	MgO smoke
Spoto *et al.*[S21]	-148	-130	FTIR spectroscopy	2004	MgO smoke
Sterrer *et al.*[S22]	-172	-155	TPD	2006	$\log(\nu) = 13$
			**Theory**		
Ugliengo *et al.*[S1]	-132	-113	Cluster MP2:B3LYP	2002	HL:LL mechanical embedding
Herschend *et al.*[S3]	-124	-105	Cluster MP2	2006	Electrostatic embedding
Qin *et al.*[S4]	-110	-91	Cluster CISD	2008	Electrostatic embedding
Staemmler[S5]	-124	-105	Cluster CEPA	2011	Method of local increments
Boese *et al.*[S6]	-218	-199	Cluster MP2+ΔCC:PBE-D2 [Ne]	2013	HL:LL mechanical embedding
Li *et al.*[S10]	-31	-12	Cluster MP2	2015	Cluster-in-molecule approach
Bajdich *et al.*[S9]	-310	-291	Periodic RPA	2015	Supercell approach
Heuser *et al.*[S12]	-408	-389	Cluster CC2	2016	Frozen-density embedding
Mazheika and	70	89	Cluster CCSD(T)	2016	Electrostatic embedding
Levchenko[S11]	-10	9	Cluster RPA(+rSE+SOSEX)	2016	Electrostatic embedding
Alessio *et al.*[S7]	-230	-211	Periodic LMP2+ΔCC	2019	HL:LL mechanical embedding
Mitra *et al.*[S13]	-398	-379	Periodic CCSD	2022	Supercell approach

S3 Benchmarking the revPBE-D4 geometry

Unfortunately, obtaining energy gradients is highly expensive for reference methods such as quantum diffusion Monte Carlo (DMC) or coupled cluster with single, double and perturbative triple particle-hole excitation operators [CCSD(T)]. As such, geometry optimizations cannot be performed with these methods and so the geometries have to be approximated with less accurate methods such as DFT. To decide on the DFT functional to provide geometries for the reference methods in this work, we have compared the adsorption energy E_{ads}, bulk MgO lattice parameter and Mg – C distance (i.e. the distance between the C atom and the closest five-coordinated Mg atom) against suitable references (discussed below). We chose to benchmark 6 high-accuracy DFT functionals: PBE-D2 [Ne], revPBE-D4, vdW-DF, rev-vdW-DF2, PBE0-D4 and B3LYP-D2 [Ne], where [Ne] denotes that the Neon D2 parameters[S23] have been used on the Mg atom.[S24]

We used the adsorption energy E_{ads} determined in Sec. S6 for the cluster CCSD(T) technique as the reference. Alongside this, we also calculated binding curves with cluster CCSD(T) of the CO molecule as a function of distance from the surface, both frozen at their revPBE-D4 geometries in the combined CO adsorbed on MgO system, to determine a reference Mg – C distance for comparison to DFT. The reference lattice parameter was taken from experiment.[S25] As shown in Table S2, we find that revPBE-D4 is the most appropriate DFT functional as it provides the best agreement to the reference E_{ads} and lattice parameter alongside reasonable Mg – C distance, all at a low computational cost.

Table S2: Comparison of the adsorption energy E_{ads}, lattice parameter and Mg – C distance between DFT and reference values (discussed in text). This has been compared for PBE-D2 [Ne], revPBE-D4, vdW-DF, rev-vdW-DF2, PBE0-D4 and B3LYP-D2 [Ne], where Ne D2 parameters were used on the Mg atom for the functionals with D2 [Ne].

	E_{ads} (meV)	Lattice Parameter (Å)	Mg – C distance (Å)
PBE-D2 [Ne]	-228	4.234	2.421
revPBE-D4	-207	4.220	2.460
vdW-DF	-232	4.273	2.544
rev-vdW-DF2	-266	4.220	2.413
PBE0-D4	-234	4.175	2.460
B3LYP-D2 [Ne]	-149	4.202	2.512
Reference	-199	4.217	2.508

S4 Uncertainty in geometrical relaxation (Δ_{geom})

As discussed in the main text, the adsorption energy E_{ads} is partitioned into an interaction energy E_{int} and geometrical relaxation term Δ_{geom}. This partition has been chosen to enable accurate calculations of E_{int} (the major term for CO on MgO adsorption). Here, the revPBE-D4 geometry for the CO adsorbed on MgO structure has been used for the E_{int} calculations involving DMC and CCSD(T). Δ_{geom} is computed directly at the revPBE-D4 level on the revPBE-D4 geometries. We have also used the Δ_{geom} term to encapsulate the errors for using the revPBE-D4 geometry on the overall E_{ads} term. To do this, we have calculated the adsorption energy with 5 other DFT functionals: PBE-D2 [Ne], vdW-DF, rev-vdW-DF2, PBE0-D4 and B3LYP-D2 [Ne]. Here, adsorption energy with the true geometry (i.e. lattice parameter and surface relaxation) arising from each of these 5 functionals is denoted E_{ads}^{True}. This is compared against the value obtained using the revPBE-D4 geometries computed at the corresponding DFT level with the revPBE-D4 Δ_{geom} (of 8 meV) to give E_{ads}^{Approx}. As shown in Table S3, the resulting E_{ads}^{Approx} is very close to E_{ads}^{True} for the 5 functionals, with an RMSD of only 10 meV.

Table S3: Comparison of the (true) adsorption energy $E_{\text{ads}}^{\text{True}}$ from using the appropriate DFT functional geometry and (approximate) adsorption energy $E_{\text{ads}}^{\text{Approx.}}$ from using the revPBE-D4 geometry and Δ_{geom}. This difference has been compared for PBE-D2 [Ne], vdW-DF, rev-vdW-DF2, PBE0-D4 and B3LYP-D2 [Ne]. All energies are given in meV.

	$E_{\text{ads}}^{\text{True}}$	$E_{\text{ads}}^{\text{Approx.}}$	Difference
PBE-D2 [Ne]	-228	-225	-4
revPBE-D4	-207	-207	
vdW-DF	-232	-212	-20
rev-vdW-DF2	-266	-265	-1
PBE0-D4	-234	-241	7
B3LYP-D2 [Ne]	-149	-148	-1
RMSD			10

S5 Computational details and convergence

S5.1 Cluster CCSD(T)

Our cluster calculations use an electrostatic embedding approach. The overall system (Fig. 1 of the main text) consists of a central quantum(-mechanically treated) cluster placed within an embedding environment. This embedding environment models the long-range electrostatic interactions of the surface by placing point charges within a 60 Å radius from the central CO molecule on the periodic 4L slab. These point charges are placed at the Mg and O ion crystallographic positions, taking formal oxidation values, with an additional outer layer of (optimized) point charges that enable the Madelung potential to be accurately represented at the quantum cluster. In the vicinity of the quantum cluster (<4 Å), the +2 point charges on the Mg ion sites are capped with the `ECP10SDF` effective core potential from the Stuttgart/Cologne group.[S26] It prevents electron leakage from the dangling bonds of the O ions in the quantum cluster boundary. These were all constructed using py-ChemShell.[S27]

The SKZCAM protocol[S28] provides the framework to generate a series of quantum clusters with rapid and systematically converging properties. For each cluster, it uses robust and chemically intuitive rubrics to first select the Mg cations, followed by the O anions. The Mg cations arrange as shells, containing symmetry-related equidistant cations around the C

atom of the CO molecule. Clusters (of only Mg cations) can be constructed by progressively incorporating more of these shells. The O anions are then placed to fully coordinate all of the Mg cations. The resulting set of clusters are always negatively charged as there will be more O anions than Mg cations; each Mg and O ion contributes +2 and −2 to the charge of the cluster.

Localized orbital correlated wave-function theory (cWFT) calculations on the quantum cluster were performed with the local natural orbital (LNO) CCSD(T)[S29–S32] and local Møller Plesset perturbation theory (LMP2)[S33] implementations of Nagy *et al.* in the MRCC[S34,S35] program suite. The very tight or "vtight" LNO local correlation thresholds were employed to closely approach conventional MP2 and CCSD(T) results. In particular, recent advancements in LNO-CCSD(T), such as its redundancy free local MP2[S33] and (T)[S29] expressions, and a CCSD(T) code[S32] redesigned specifically for the LNO method make it uniquely efficient and memory economic for large systems[S31,S36] including also ionic and surface interactions.[S28,S37] We included the sub-valence correlation of the 2s and 2p electrons on the Mg atom with the corresponding cc-pwCVnZ[S38,S39] basis set. For all other atoms, we used the aug-cc-pVnZ[S40] basis set. The def2-QZVPP-RI-JK basis set was used as the auxiliary basis function for the Hartree–Fock (HF) computations. The RI auxiliary basis sets[S41,S42] corresponding to the AO basis sets were used for the local cWFT calculations but the automatic auxiliary basis functions of Stoychev *et al.*[S43,S44] were generated for the Mg basis sets. Complete basis set (CBS) extrapolation parameters for the TZ and QZ pair, CBS(TZ/QZ), taken from Neese and Valeev,[S45] were used for the HF and correlation energy components of the cWFT total energy.

S5.2 Periodic CCSD(T)

The periodic coupled cluster theory calculations are performed using the Cc4s code,[S46] which is interfaced to the Vienna *ab initio* simulation package (VASP).[S47] The calculations are performed in several steps involving Hartree–Fock and MP2 theory to obtain corresponding

energies and optimized approximate natural orbitals.[S48] Once the natural orbitals have been computed, the Cc4s interface to VASP is used to compute intermediate quantities[S49] that are needed for the subsequent coupled cluster energy calculations including the corresponding finite size[S50] and basis set corrections.[S51] As discussed in Sec. S6, the final E_{ads} with periodic CCSD(T) is dominated mainly by the CCSD(T) interaction energy on a 2L MgO slab ($E_{\mathrm{int,2L}}^{\mathrm{CCSD(T)}}$). In Table S4, we show the key contributions that make up $E_{\mathrm{int,2L}}^{\mathrm{CCSD(T)}}$. Besides a HF ($E_{\mathrm{int,2L}}^{\mathrm{HF}}$), CCSD correlation ($E_{\mathrm{int,2L}}^{\mathrm{CCSD}}$), and perturbative triples correlation ($E_{\mathrm{int,2L}}^{\mathrm{(T)}}$) contribution to the $E_{\mathrm{int,2L}}^{\mathrm{CCSD(T)}}$ interaction energy, there are also finite size corrections and basis set incompleteness error correction, denoted as $\Delta_{\mathrm{FS}}^{\mathrm{CCSD}}$ and $\Delta_{\mathrm{FPC}}^{\mathrm{CCSD}}$ respectively. In Ref. S52, all individual steps are described when combined with an embedding approach (which was not employed for the present system)

Table S4: The contributions making up $E_{\mathrm{int,2L}}^{\mathrm{CCSD(T)}}$. All energies given in meV.

Method	Comp. details	$E_{\mathrm{int,2L}}$
$E_{\mathrm{int,2L}}^{\mathrm{CCSD(T)}}$		-182
$E_{\mathrm{int,\,2L}}^{\mathrm{HF}}$	1×1×1 k-mesh	41
$E_{\mathrm{int,\,2L}}^{\mathrm{CCSD\ corr.}}$	10 NOs/occ., 1×1×1 k-mesh	-159
$\Delta_{\mathrm{FS,\,2L}}^{\mathrm{CCSD}}$	10 NOs/occ., 1×1×1 k-mesh	-18
$\Delta_{\mathrm{FPC,\,2L}}^{\mathrm{CCSD}}$	10 NOs/occ., 1×1×1 k-mesh	2
$E_{\mathrm{int,\,2L}}^{\mathrm{(T)\ corr.}}$	10 NOs/occ., 1×1×1 k-mesh	-48

We employ the PAW POTCAR files labeled as `C_GW`, `Mg_pv_GW` (corresponding to 8 valence electrons on the Mg) and `O_GW_new` for calculating $E_{\mathrm{int,2L}}^{\mathrm{CCSD(T)}}$. We chose a plane-wave cutoff parameter of `ENCUT` $= 500\,\mathrm{eV}$. If not stated otherwise, a Γ-centered 1×1×1 k-mesh is used to sample the first Brillouin zone. These contributions are obtained using 10 unoccupied natural orbitals per occupied orbital. For the present system containing the CO molecule adsorbed on the 2L MgO surface, this corresponds to 117 occupied spatial orbitals and 1170 unoccupied spatial orbitals. The computational cost for a CCSD(T) calculation of the adsorbed CO system including an MP2, CCSD and (T) step is about 2 kCPUh, 6 kCPUh and 80 kCPUh, respectively. The memory requirements are also significant; the CCSD and

(T) calculations require about 3 TB of distributed memory. It should be noted that $\Delta_{\text{FPC}}^{\text{CCSD}}$ depends on estimates of the MP2 pair energies, which are computed using 100 natural orbitals per occupied state and an automated extrapolation to the CBS limit.

We have checked convergence of the computed interaction energies with respect to all employed parameters and found that the only significant uncertainty originates from the basis set incompleteness error in the estimates of $E_{\text{int, 2L}}^{\text{CCSD corr.}} + \Delta_{\text{FPC, 2L}}^{\text{CCSD}}$. Estimating the remaining basis set incompleteness error requires calculations with larger basis sets. We have computed the change in $E_{\text{int, 2L-flat}}^{\text{CCSD corr.}} + \Delta_{\text{FPC, 2L-flat}}^{\text{CCSD}}$ when employing 15 instead of 10 natural orbitals per occupied state for a perfect flat surface (i.e. bulk truncated) in the initial phase of this project. Our findings in Table S5 show that this changes the interaction energy by 22 meV. Due to the observed rapid convergence of $E^{\text{CCSD corr.}} + \Delta_{\text{FPC}}^{\text{CCSD}}$ with respect to the number of natural orbitals reported for a wide range of molecular benchmark systems in Ref. S51, we conclude that ±22 meV is a very conservative estimate of the CCSD contributions to the interaction energy reported in this work. The other potential source of error is that the CO molecule in the unbound structure has only been shifted by 5 Å w.r.t. the bound structure, but at the rev-vdW-DF2 level, we find that this changes $E_{\text{int,4L}}$ by only 5 meV as opposed to the first definition of E_{int} in Eq. 2 of the main text, equivalent to an infinite shift.

Table S5: The change in the CCSD interaction energy combined with basis set corrections ($E_{\text{int, 2L-flat}}^{\text{CCSD corr.}} + \Delta_{\text{FPC, 2L-flat}}^{\text{CCSD}}$) against number of natural orbitals per occupied state (NOs/occ) for CO adsorbed on a flat (non-corrugated) 2L MgO surface. The difference w.r.t. the largest 15 NOs/occ calculation is given for the smaller basis sets. All energies given in meV.

NOs/occ	$E_{\text{int, 2L-flat}}^{\text{CCSD corr.}} + \Delta_{\text{FPC, 2L-flat}}^{\text{CCSD}}$	Diff
5	-205	-46
10	-137	22
15	-159	0

S5.3 Periodic DMC

All calculations were performed in CASINO.[S53] We used ccECP pseudopotentials[S54,S55] for all of the atoms. The Jastrow factor included two-body electron–electron (e–e) term, two-body electron–nucleus (e–n) terms, and three-body electron–electron–nucleus (e–e–n) terms. These variational parameters were optimized by minimizing the variance for the bound structure (either 2L for the 10 valence electron Mg ccECP or 4L for the 2 valence electron Mg ccECP), with the Jastrow subsequently used for all other systems, whether unbound or bound. PWSCF[S56] was used to initialize the wavefunction using the LDA functional. We ensured the convergence of the timestep τ for both the 10 electron and 2 electron Mg ccECP. As seen in Table S6, the 2 electron pseudopotential was already converged at $\tau = 0.1$. On the other hand, the 10 electron ccECP requires a smaller timestep of $\tau = 0.03$. We incorporate the model periodic coulomb (MPC) correction[S57–S59] for a shorter duration than with plain Ewald interaction because it is very expensive (around $6\times$ more). Fortunately, its correction up from Ewald requires significantly fewer steps to reach the same statistical errors. We note that the results in Table S6 do not include this correction (and other contributions discussed in Sec. S6), hence the lower value than shown in the main paper. All quoted statistical errors are given as two standard deviations (2σ) in the main text.

Table S6: The convergence of the DMC interaction energy E_{int} in meV with timestep (0.1, 0.03 and 0.01 au) for the 2 valence electron Mg ccECP and 10 valence electron Mg ccECP on the 4L and 2L slabs respectively. These calculations were performed without the MPC correction.

	0.1	0.03	0.01
4L (2 electron Mg PP)	-142 ± 18	-144 ± 19	
2L (10 electron Mg PP)	-140 ± 22	-159 ± 14	-161 ± 25

S5.4 DFT

Periodic supercell calculations with DFT were performed in the Vienna *Ab initio* Simulation Package (VASP).[S47,S60] The (001) MgO surface calculations employed an asymmetric four-layer slab with the top two layers allowed to relax to form the pristine surface for a (4×4) supercell, with 15 Å of vacuum between the two surfaces and $(2\times2\times1)$ Γ-centred Monkhorst-Pack k-point sampling. We chose a plane-wave cutoff parameter of `ENCUT` $= 600$ eV., with the 8 valence electron `Mg_pv` PAW potential used for Mg, while standard PAW potentials were used for all other atoms. As shown in Table. S7, we confirmed that all of these (a) chosen settings are converged to within 3 meV by comparing against (b) a larger supercell, (c) more slab layers and (d) more expensive electronic structure settings, as shown in Table. S7.

Table S7: The change in E_{ads} of the revPBE-D4 DFT adsorption energy E_{ads} for the (a) 4×4 supercell with 4L slab as the (b) supercell size is increased, (c) layer number is increased and (d) energy cutoff, grid precision and k-point density is increased.

Label	Supercell Size	Number of Layers	K-point Mesh	Energy Cutoff	PREC	E_{ads} (meV)	Change (meV)
(a)	4×4	4	$2 \times 2 \times 1$	600	Normal	-206	0
(b)	6×6	4	$2 \times 2 \times 1$	600	Normal	-203	3
(c)	4×4	6	$2 \times 2 \times 1$	600	Normal	-207	-1
(d)	4×4	4	$3 \times 3 \times 1$	700	Accurate	-206	1

S6 Details on E_{ads} estimates and computational costs

As shown in Table S8, the final E_{ads} for each of the three techniques [cluster CCSD(T), periodic CCSD(T) and periodic DMC] is composed of several contributions, with Δ_{geom} having been discussed previously. Here, we have attempted to make highly conservative estimates of the error bars to the most important contributions for all three approaches. The root sum square of all these errors were then taken as the final error for each method, as the individual error contributions are assumed to be uncorrelated.

For similar reasons to the cluster CCSD(T) technique (discussed in the main text and Sec. S7), this decomposition has been made because it is not possible to directly compute E_{int} accurately at the converged 4L (4×4) slab with either DMC or CCSD(T). For both methods, we have only been able to apply them to a smaller 2L slab, which was cleaved from the original 4L slab. This contribution forms the majority of the final E_{ads} for both CCSD(T) and DMC, given by $E_{\text{int,2L}}^{\text{CCSD(T)}}$ and $E_{\text{int,2L}}^{\text{DMC}}$ respectively. In the vein of Pople's model chemistry,[S61] we have computed the remaining (much smaller) contributions with more computationally economical methods. A pseudopotential (PP) is utilized for both periodic calculations, being either a PAW potential for periodic CCSD(T) or an effective core potential (from the ccECP family[S54,S55]) for periodic DMC. Here, the number of valence electrons associated with the Mg PP is another controllable factor which affects the accuracy and cost. In particular, we show in Sec. S8 that besides the 3s, inclusion of 2s and 2p electrons to form a 10 valence electron Mg PP is necessary to match all-electron calculations.

For DMC, we used a more economical form of DMC as the lower level, hereafter referred to as LL-DMC, that uses a 2 valence electron Mg PP, which (i) results in fewer electrons and (ii) allows for a larger timestep of $\tau = 0.1$ that remains converged (as shown in Sec. S5.3). The 10 valence electron Mg PP DMC calculation on the 2L MgO slab $\left(E_{\text{int,2L}}^{\text{DMC}}\right)$ was corrected up to a 4L MgO slab in the $\Delta_{\text{2L} \rightarrow \text{4L}}^{\text{LL-DMC}}$ term with LL-DMC. We also used LL-DMC account for finite size errors (FSE) with the $\Delta_{\text{FSE}}^{\text{LL-DMC}}$ term using a Model Periodic Coulomb (MPC) correction[S57–S59] in the 4L MgO slab. The final (small) $\Delta_{\text{IPFSE}}^{\text{LDA}}$ contribution is a correction

Table S8: Comparison of the final E_{ads} estimates between the cluster CCSD(T), periodic CCSD(T) and periodic DMC techniques. We give the walltime in kCPU-hours and maximum RAM usage in GB. No RAM usage has been given for DMC because it uses a negligible amount relative to CCSD(T). The components making up the final estimates are also shown (see text for description). Here, conservative estimates have been made for the errors of key components, which we discuss in Secs. S5, S6 and S7. The final error in E_{ads} is taken as the root sum square of these errors.

Cluster CCSD(T)	Contribution (meV)	Cost (kCPUh)	RAM (GB)
Final E_{ads}	$\mathbf{-199 \pm 11}$	~ 20	~ 20
$E^{\text{MP2}}_{\text{int,bulk-lim}}$	-200 ± 5		
ΔCC	-7 ± 3		
Δ_{geom}	8 ± 10		
**Periodic CCSD(T)**	**Contribution (meV)**	**Cost (kCPUh)**	**RAM (GB)**
Final E_{ads}	$\mathbf{-193 \pm 24}$	~ 200	~ 3000
$E^{\text{CCSD(T)}}_{\text{int,2L}}$	-182 ± 22		
$\Delta^{\text{MP2}}_{\text{2L} \to \text{4L}}$	-6		
$\Delta^{\text{MP2}}_{\text{core, 4L}}$	-8		
$\Delta^{\text{HF}}_{\text{IPFSE}}$	-5		
Δ_{geom}	8 ± 10		
**Periodic DMC**	**Contribution (meV)**	**Cost (kCPUh)**	**RAM (GB)**
Final E_{ads}	$\mathbf{-188 \pm 26}$	~ 1000	N/A
$E^{\text{DMC}}_{\text{int,2L}}$	-159 ± 14		
$\Delta^{\text{LL-DMC}}_{\text{2L} \to \text{4L}}$	-9 ± 19		
$\Delta^{\text{LL-DMC}}_{\text{FSE}}$	-33 ± 5		
$\Delta^{\text{LDA}}_{\text{IPFSE}}$	5		
Δ_{geom}	8 ± 10		

for Independent Particle FSE[S62] (IPFSE) computed at the LDA level going from a $1 \times 1 \times 1$ Gamma-centered k-point mesh to a $3 \times 3 \times 1$ mesh. Besides the final term, which is very small, statistical (2σ) error bars can be estimated for all of the above terms, which are expected to be much bigger than the systematic errors arising from using a lower level theory.

In periodic CCSD(T), we use MP2, CCSD and Hartree-Fock (HF) as lower level theories. As discussed in Sec. S5.2, the 2L CCSD(T) interaction energy $E^{\text{CCSD(T)}}_{\text{int,2L}}$ has been computed with an 8 valence electron Mg PP (the `Mg_pv_GW` POTCAR in VASP), with two subsequent corrections using MP2 up to (i) a 4L MgO slab $\Delta^{\text{MP2}}_{\text{2L} \to \text{4L}}$ and (ii) a 10 electron Mg PP $\Delta^{\text{MP2}}_{\text{core, 4L}}$. For the former correction, we have estimated $E^{\text{MP2}}_{\text{int}}$ for the 2L and 4L systems to give $\Delta^{\text{MP2}}_{\text{2L} \to \text{4L}}$. For the latter correction, we have employed the POTCAR file labeled `Mg_sv_GW`

with 10 valence electrons and the difference in $E_{\text{int}}^{\text{MP2}}$ between `Mg_sv_GW` and `Mg_pv_GW` is estimated for the 4L slab to give $\Delta_{\text{core, 4L}}^{\text{MP2}}$. The periodic MP2 calculations here used 50 natural orbitals per occupied state. Finally, IPFSE is accounted for in $\Delta_{\text{IPFSE}}^{\text{HF}}$ at the HF level estimated using the difference between $E_{\text{int}}^{\text{HF}}$ computed with a 1×1×1 and 2×2×1 k-mesh for the 4L slab. It can be seen in Table S8 that the most dominant term to the final periodic CCSD(T) E_{ads} is $E_{\text{int,2L}}^{\text{CCSD(T)}}$ and as discussed in Sec. S5.2, its main source of error lies in basis set incompleteness errors, which we provide a conservative estimate of 22 meV. The other terms are significantly smaller and expected to contribute negligible errors relative to $E_{\text{int,2L}}^{\text{CCSD(T)}}$.

We discuss the details of the individual terms to the cluster CCSD(T) E_{ads} in the next section.

S7 SKZCAM protocol and its convergence

As discussed in the main text, the final estimated adsorption energy E_{ads} arising from the cluster CCSD(T) technique was composed of an MP2 contribution $E_{\text{int,bulk-lim}}^{\text{MP2}}$ followed by a correction up to CCSD(T), termed ΔCC. These quantities can be computed accurately and in a cheap manner using the SKZCAM protocol and LNO-CCSD(T).[S28] Here, this protocol produces a set of clusters of systematically increasing size, for which we can compute the interaction energy E_{int} at several levels of theory (MP2, CCSD(T) and their local variants) at different basis sets. In Table S9, we compute the E_{int} for several levels of theory along some of the clusters generated by the SKZCAM protocol and describe in the next two subsections how $E_{\text{int,bulk-lim}}^{\text{MP2}}$ and ΔCC are estimated.

S7.1 Extrapolation to MP2 bulk limit

The smooth convergence with cluster size (see Fig. 2b of the main text) allows for the bulk limit to be estimated via an extrapolation. We used the following formula for the relationship

Table S9: The interaction energy E_{int} (in meV) computed along the series of clusters produced from the SKZCAM protocol[S28] for the CO on MgO system. We provide estimates for MP2 with double-zeta (DZ), triple-zeta (TZ) and quadruple-zeta (QZ) basis functions (see Sec. S5). The complete basis set (CBS) limit for the TZ and QZ pair is computed for MP2 and canonical CCSD(T), while the CBS limit for the DZ and TZ pair is computed for local MP2 (LMP2) and LNO-CCSD(T).

		MP2					CCSD(T)	
Cluster	# of atoms	DZ	TZ	QZ	CBS	Local CBS	Local (LNO) CBS	Canonical CBS
1	6	-45	-119	-140	-153	-154	-159	-160
2	22	-99	-155	-173	-184	-184	-191	
3	34	-110	-164	-178	-188	-187	-195	
4	42	-116	-168	-182	-192			
5	58	-122	-171	-184	-193			
6	82	-127						
7	84	-128						
8	100	-131						

between a cluster of size N atoms and E_{int}:

$$E_{\text{int}}[N] = A + \frac{B}{N^{\gamma}}, \tag{1}$$

where A, B and γ are parameters to be fit via linear regression, with A being the bulk limit $E_{\text{int, bulk-lim}}$. In Table S10, we show the estimate of $E_{\text{int, bulk-lim}}$ as more clusters are included in the linear regression fit at the DZ and CBS(TZ/QZ) basis sets at the MP2 level; QZ calculations were only feasible up to 5 clusters if we wanted to stay within a 2 day walltime on a single node. An estimate on the errors for only including 5 clusters in the MP2 bulk limit extrapolation can be estimated from observing the convergence of the DZ basis set, for which calculations can be performed on much larger clusters. As seen in Table S10, the difference for using 5 clusters w.r.t. the converged value (from extrapolating 8 clusters) is only 5 meV, which we take as an estimate of its error in $E_{\text{int, bulk-lim}}$ in the main text; this error is expected to be a conservative estimate because the convergence with the DZ basis set is slower than CBS(TZ/QZ).

Table S10: The extrapolated bulk limit interaction energy $E_{\text{int, bulk-lim}}$ at the MP2 level for the DZ and CBS(TZ/QZ) basis sets as we include more clusters (from the SKZCAM protocol) in the extrapolation. The error w.r.t. to the largest set of clusters (8) is also computed for the DZ basis set. All energies are given in meV.

Cluster	DZ	Error	CBS
3	-156	4	-194
4	-174	15	-202
5	-164	5	-200
6	-157	3	
7	-158	1	
8	-159		

S7.2 ΔCC correction

The steep cost of canonical CCSD(T)/CBS means that it can only be performed on the smallest one or maybe two clusters generated by the SKZCAM protocol. With local approximations, such as with LNO-CCSD(T), the feasible cluster sizes which can be studied are significantly extended. Additionally, the computation of accurate bulk-limit (extrapolated) CCSD(T) estimates can be formulated efficiently by adding the difference between the CCSD(T) and MP2 E_{int} estimates (ΔCC correction), to $E_{\text{int,bulk-lim}}^{\text{MP2}}$. Since well-converged ΔCC corrections can be obtained using only the first few clusters of the SKZCAM protocol, the corresponding individual LNO-CCSD(T) computations become very economical. For example, it required less time on a single (many-core) computer node than the typical 1–2 days wall time limits accessible in commodity high-performance computing clusters.

As seen in Table S11, the chosen vtight LNO settings gives LMP2 estimates that are within 1 meV w.r.t. canonical MP2 for the first three clusters. This was also confirmed between LNO-CCSD(T) and canonical CCSD(T) for the first cluster. Importantly, we find that we do not need to go beyond the first three clusters of the SKZCAM protocol with LNO-CCSD(T) or LMP2 because the $\Delta\text{CC}^{\text{local}}$ difference between LNO-CCSD(T) and LMP2 stays consistent to within 3 meV across these three clusters. We have chosen to make ΔCC the average $\Delta\text{CC}^{\text{local}}$ from these three clusters with an error bar of 3 meV (in Sec. S6)

corresponding to the largest observed deviation across these clusters.

Table S11: The interaction energy E_{int} at the local MP2, LNO-CCSD(T), (canonical) MP2 and (canonical) CCSD(T) levels. The ΔCC correction is computed for both the local and canonical levels of theory. The complete basis set (CBS) limit for the TZ and QZ pair is computed for MP2 and canonical CCSD(T), while the CBS limit for the DZ and TZ pair is computed for local MP2 (LMP2) and LNO-CCSD(T). All energies are given in meV.

Cluster	LMP2	LNO-CCSD(T)	$\Delta\text{CC}^{\text{local}}$	MP2	CCSD(T)	ΔCC
1	-154	-159	-5	-153	-160	-7
2	-184	-191	-7	-184		
3	-187	-195	-8	-188		

S8 Analysis of prior CO on MgO simulations

The differences in our estimated values of E_{ads} w.r.t. previous simulations can be identified to arise from factors such as: (i) frozen core size, (ii) basis set size, (iii) basis set superposition error (BSSE) and (iv) use of unconverged cluster sizes. If all of these settings are accounted for, then any differences can be attributed to the inadequacy of the chosen level of theory.

Table S9 shows that using a smaller (ii) basis set leads to a weaker binding (i.e. a less negative E_{int}) compared to the CBS limit, assuming counterpoise corrections. Assuming no counterpoise corrections (CPC) to fix for (iii) BSSE, we find that the E_{int} strongly overbinds, predicting a MP2 CBS(DZ/TZ) E_{int} of -294 meV for the 5th cluster relative to -196 meV when CPC is included. For this 5th cluster, we have also found that a larger (i) frozen core (i.e. only correlating valence 3s but not 2s or 2p electrons) on the Mg causes a weaker binding, giving E_{int} of -165 meV. Finally, using an unconverged cluster size also typically causes weaker binding, as already demonstrated in Table S9, where E_{int} becomes more negative (i.e. exothermic) with larger clusters. Many embedded cluster studies specifically use the cubic $3 \times 3 \times 2$ (Mg_9O_9) cluster in their studies and it also provides weaker binding, giving a CBS(TZ/QZ) binding energy of -184 meV at the MP2 level relative to the final estimated value of -200 meV in the CBS(TZ/QZ) bulk limit.

We have assessed many of the previous simulations against these four factors in Table S12 and have been able to understand their trends relative to our converged E_{ads} estimates. For example, the three early studies by Ugliengo *et al.*,[S1] Herschend *et al.*[S3] and Qin *et al.*[S4] all do not include 2s and 2p electrons in their correlation treatment of the Mg atom while using small unconverged basis sets, leading to a weakening of the binding. The study by Staemmler *et al.*[S5] so far seem to have converged all four factors, probably suggesting that the CEPA method used is inadequate; it is formally based upon CCSD, which is a method that produces weaker binding than CCSD(T) for CO on MgO.[S6] The studies by Boese *et al.* and Alessio *et al.* both agrees with our estimated values to within about 20–30 meV because their studies have confirmed the sufficient convergence of all four factors. The study by Mitra *et al.* as well as by Heuser *et al.*[S12] strongly overbinds the E_{ads} because they have not included CPC to account for the BSSE. Finally, the studies by Li *et al.*[S10] and Mazheika and Levchenko[S11] predict weaker binding because they use a smaller unconverged basis set, with Mazheika and Levchenko predicting the weakest binding (in fact repulsion), because it uses a small cluster and does not include 2s and 2p electrons in the correlation treatment.

Table S12: Table indicating whether the (i) Frozen core, (ii) Basis set, (iii) Basis superposition error and (iv) Cluster size have been converged or corrected in the past simulation work. We also include here their values of E_{ads} from Table S1, which can be compared to our fully converged cluster CCSD(T) value of -199 ± 11 meV.

Study	E_{ads} (meV)	Frozen core	Basis set	BSSE	Cluster size
Ugliengo *et al.*[S1]	−132	No	Yes	Yes	No (Mg_9O_9)
Qin *et al.*[S4]	−110	No	No (DZ)		No (Mg_9O_9)
Herschend *et al.*[S3]	−124	No	No (TZ)	Yes	Yes ($Mg_{18}O_{18}$)
Staemmler[S5]	−124	Yes	Yes	Yes	Yes
Boese *et al.*[S6]	−218	Yes	Yes	Yes	Yes
Alessio *et al.*[S7]	−230	Yes	Yes	Yes	Yes
Li *et al.*[S10]	−31	N/A	No [6-311+G(2df)]	N/A	Yes
Heuser *et al.*[S12]	−408	N/A	No (TZ)	No	No (Mg_9O_9 w/o embedding)
Mazheika and Levchenko[S11]	70	No	No (DZ & TZ)	Yes	No (Mg_9O_9)
Mitra *et al.*[S13]	−398	Yes	No (TZ)	No	Yes (supercell)

S9 Reaching accurate experimental E_{ads}

The low (and well-controlled) errors of the cluster CCSD(T) E_{ads} provides the opportunity to evaluate the previous experimental work in detail. We focus particularly on the recent 5 experiments, involving three temperature programmed desorption (TPD) and two Fourier transform infrared (FTIR) experiments; the inaccuracies (and large variations) of earlier work has been discussed in detail before.[S21,S63–S65] As seen in Fig. S1, the TPD experiments can all reach closer agreement to the cluster CCSD(T) value than FTIR, with both FTIR E_{ads} estimates (given by orange markers), performed by Spoto *et al.*,[S20,S21] significantly underestimating the cluster CCSD(T) numbers. This underestimation can be attributed to two uncontrolled approximations used in the FTIR analysis: (i) IR intensities are proportional to CO coverage and (ii) H_{ads} is independent of coverage. Due to dipole-dipole interactions between CO molecules, H_{ads} decreases with CO coverage on many surfaces (including MgO) and the FTIR assumptions would thus result in smaller (i.e. less negative) H_{ads}.

There are fewer assumptions involved with the three TPD estimates by Wichtendahl *et al.*,[S16] Dohnálek *et al.*[S17] and Sterrer *et al.*[S22] As a result, there is excellent agreement between the three TPD experiments and our cluster CCSD(T) estimate (Fig. S1). This agreement is made possible by converting the TPD activation energy E_{act} quoted in the original literature to an adsorption energy E_{ads} (with an intermediate step to convert it to an adsorption enthalpy H_{ads}) as shown in Fig. S1b and discussed in more detail below. We also explain why we expect the studies by Wichtendahl *et al.* and Dohnálek *et al.* to be more accurate than the one by Sterrer *et al.*. Hence, this is why the average of these two experiments forms the (best) experimental estimate given in Fig. 1 of the main text (-198 ± 19 meV).

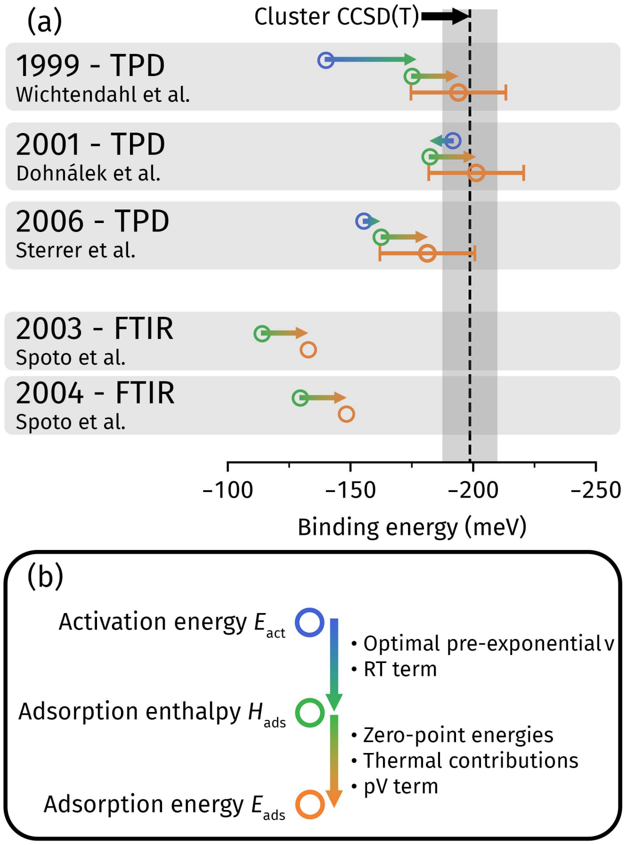

Figure S1: (a) Comparison of recent temperature programmed desorption (TPD) and Fourier-transform infrared (FTIR) spectroscopy experiments on the adsorption energy of CO on MgO. These values were compared to the reference cluster CCSD(T) calculation from this work and we find that the TPD experiments from Dohnálek *et al.*[S17] and Wichtendahl *et al.*[S16] obtained the closest agreement. (b) This agreement required accounting for several effects to convert the original activation energies (from TPD experiments) to appropriate adsorption energies which we discuss in the text.

S9.1 Converting TPD $E_{\mathbf{act}}$ to $H_{\mathbf{ads}}$

As discussed by both Campbell and Sellers[S66] as well as Sauer,[S65] Arrhenius activation energies E_{act} are predicted by TPD experiments and converting to an adsorption enthalpy H_{ads} requires adding a $\frac{1}{2}$RT term to E_{act} (i.e. $H_{\text{ads}} = E_{\text{act}} + \frac{1}{2}\text{RT}$ in the convention that E_{act} is negative for binding).

We also discuss here the past inaccuracies of the pre-exponential factor ν arising in the Redhead equation. Most studies (e.g. Wichtendahl *et al.*[S16] and Sterrer *et al.*[S22]) simply assign it a value of $\log(\nu) = 13$, which is well known to typically underestimate the true value. The study by Dohnálek *et al.*[S17] attempted to obtain this value, coming to an estimate of $\log(\nu) = 15$, but with very large error bars of ± 2. Campbell and Sellers[S66] were able to provide a value of 13.8 ± 1.6 (with 2σ error bars), which is, to date, the most reliable estimate of $\log(\nu)$. As shown by the original work from Redhead,[S67] the dependence of E_{act} on ν is of the form: $E_{\text{act}} = R\text{T}_p \ln(\nu) + A$, where A is a constant and T_p is the temperature of the CO desorption peak. Thus a factor 10 change in ν causes a $\sim 2.3\text{RT}_p$ change in E_{act}. We performed this correction to the accurate ν value for the study by Sterrer *et al.*. It has already been performed by Campbell and Seller for the studies by Wichtendahl *et al.* and Dohnálek *et al.* and we simply use their derivation.

S9.2 Converting $H_{\mathbf{ads}}$ to $E_{\mathbf{ads}}$

The adsorption enthalpy H_{ads} and the adsorption energy E_{ads} can be converted between one another through the equation:

$$E_{\text{ads}} = H_{\text{ads}}(T) - \Delta_{\text{ZPV}} - \Delta_{\text{th}}(T) + \text{RT} = H_{\text{ads}}(T) - E_{\text{cor}}. \tag{2}$$

Here, $\Delta_{\text{th}}(\text{T})$ is the thermal energy contribution at temperature T, Δ_{ZPV} is the zero point vibrational energy effects and the RT term arises due to pV volume work. For $\Delta_{\text{th}}(\text{T})$, we set T to 61K, the average desorption temperature of CO at low coverage, taken from Campbell

and Sellers[S66] These three terms form a correction term which we call E_{cor}. The Δ terms represent differences in ZPV or thermal energy contributions between the molecule from the combined molecule-surface system (with the surface degrees of freedom frozen) and the gas-phase molecule.

We used the quasi-rigid rotor harmonic oscillator approximation (quasi-RRHO) to compute both Δ_{ZPV} and $E_{\mathrm{th}}(\mathrm{T})$. These were first proposed by Grimme *et al.*[S68] for entropies and then adapted for enthalpies by Li *et al.*[S69] We leave the description of the details to these two references. In Table S13, we have calculated the Δ_{ZPV}, $\Delta_{\mathrm{th}}(\mathrm{T})$ and RT contributions to the E_{cor} for the set of 6 DFT functionals (PBE-D2 [Ne], revPBE-D4, vdW-DF, rev-vdW-DF2, PBE0-D4 and B3LYP-D2 [Ne]) that we have used throughout this study. On the MgO surface, the surface degrees of freedom have been fixed. The average E_{cor} value was the average taken from all 6 DFT functionals. There was only a standard deviation of 1 meV, suggesting E_{cor} is not particularly dependent on the DFT functional, despite E_{ads} depending significantly (Table S2). The resulting E_{cor} of 19 meV is in agreement with Boese *et al.*[S6] As discussed in their work, the anharmonic effects are expected to be relatively small compared to the errors intrinsic in experimental estimates.

Table S13: The contributions (Δ_{ZPE}, Δ_{th} and RT) that make up E_{cor} for the 6 different functionals. All energies are given in meV.

	Δ_{ZPE}	$\Delta_{\mathrm{th}}(\mathrm{T})$	RT	E_{cor}
PBE-D2 [Ne]	31	-6	5	20
revPBE-D4	28	-5	5	18
vdW-DF	28	-5	5	17
rev-vdW-DF2	32	-6	5	21
PBE0-D4	30	-5	5	19
B3LYP-D2 [Ne]	29	-5	5	18

S9.3 Comparing final experimental estimates

For clarity, we give the final TPD estimates (with ν corrected) in Table S14 below.

Table S14: The adsorption energy E_{ads} for the temperature programmed desorption experiments with ν corrected. The CO surface coverage of the separate studies is also given in terms of monolayer (ML) coverage .

Reference	Eads (meV)	CO Coverage
Wichtendahl et al.	-194	low 0.3 ML coverage
Dohnalek et al.	-201	low 0.3 ML coverage
Sterrer et al.	-181	high CO coverage

Both the works of Wichtendahl *et al.* (coverage of ∼0.3 monolayer (ML)); and Dohnálek *et al.* (coverage of ∼0.3 ML) reach sufficiently low coverage where the adsorption energy is expected to be close to the dilute limit. As shown in Table S15, a coverage of 0.25 ML is only 1 meV away from the result of 0.125 ML. We note here that the reason why Sterrer *et al.* has an E_{ads} that is less negative (i.e. weaker binding) than the other two TPD studies is because it quotes a value at relatively high CO coverage, close to a full monolayer (1 ML). As shown by Dohnálek *et al.*,[S17] this is expected to cause a strong weakening in E_{ads} the closer the CO coverage gets to 1 ML. For this reason, we consider the TPD studies by Wichtendahl *et al.* and Dohnálek *et al.* to give the most accurate E_{ads} that reproduce the dilute limit. The final experimental E_{ads} value in Fig. 1 of the main text (-198 ± 19 meV) is taken to be the average of the two studies, with the error bar taken to arise from the 2σ error in ν discussed in Sec. S9.1.

Table S15: The change in CO on MgO adsorption energy E_{ads} with CO coverage. These values have been computed at the rev-vdW-DF2 level and are quoted in meV.

Supercell Size	CO coverage	Eads	Diff
$4 \times 4 \times 1$	0.125	-266	0
$2 \times 2 \times 1$	0.250	-265	1

References

(S1) Ugliengo, P.; Damin, A. Are Dispersive Forces Relevant for CO Adsorption on the MgO(001) Surface? *Chem. Phys. Lett.* **2002**, *366*, 683–690.

(S2) Chung, L. W.; Sameera, W. M. C.; Ramozzi, R.; Page, A. J.; Hatanaka, M.; Petrova, G. P.; Harris, T. V.; Li, X.; Ke, Z.; Liu, F.; Li, H.-B.; Ding, L.; Morokuma, K. The ONIOM Method and Its Applications. *Chem. Rev.* **2015**, *115*, 5678–5796.

(S3) Herschend, B.; Baudin, M.; Hermansson, K. Influence of Substrate Dynamics on CO–MgO(001) Bonding–Using Molecular Dynamics Snapshots in Quantum-Chemical Calculations. *J. Phys. Chem. B* **2006**, *110*, 5473–5479.

(S4) Qin, C. CI Study of CO Adsorption on MgO(100). *Chem. Phys. Lett.* **2008**, *460*, 457–460.

(S5) Staemmler, V. Method of Local Increments for the Calculation of Adsorption Energies of Atoms and Small Molecules on Solid Surfaces. 2. CO/MgO(001). *J. Phys. Chem. A* **2011**, *115*, 7153–7160.

(S6) Boese, A. D.; Sauer, J. Accurate Adsorption Energies of Small Molecules on Oxide Surfaces: CO–MgO(001). *Phys. Chem. Chem. Phys.* **2013**, *15*, 16481–16493.

(S7) Alessio, M.; Bischoff, F. A.; Sauer, J. Chemically Accurate Adsorption Energies for Methane and Ethane Monolayers on the MgO(001) Surface. *Phys. Chem. Chem. Phys.* **2018**, *20*, 9760–9769.

(S8) Pisani, C.; Maschio, L.; Casassa, S.; Halo, M.; Schütz, M.; Usvyat, D. Periodic Local MP2 Method for the Study of Electronic Correlation in Crystals: Theory and Preliminary Applications. *J. Comp. Chem.* **2008**, *29*, 2113–2124.

(S9) Bajdich, M.; Nørskov, J. K.; Vojvodic, A. Surface Energetics of Alkaline-Earth Metal

Oxides: Trends in Stability and Adsorption of Small Molecules. *Phys. Rev. B* **2015**, *91*, 155401.

(S10) Li, W.; Chen, C.; Zhao, D.; Li, S. LSQC: Low Scaling Quantum Chemistry Program. *Int. J. Quantum Chem.* **2015**, *115*, 641–646.

(S11) Mazheika, A.; Levchenko, S. V. Ni Substitutional Defects in Bulk and at the (001) Surface of MgO from First-Principles Calculations. *J. Phys. Chem. C* **2016**, *120*, 26934–26944.

(S12) Heuser, J.; Höfener, S. Wave-Function Frozen-Density Embedding: Approximate Analytical Nuclear Ground-State Gradients. *J. Comp. Chem.* **2016**, *37*, 1092–1101.

(S13) Mitra, A.; Hermes, M. R.; Cho, M.; Agarawal, V.; Gagliardi, L. Periodic Density Matrix Embedding for CO Adsorption on the MgO(001) Surface. *J. Phys. Chem. Lett.* **2022**, *13*, 7483–7489.

(S14) Furuyama, S.; Fujii, H.; Kawamura, M.; Morimoto, T. Physisorption of Nitric Oxide, Carbon Monoxide, Nitrogen, and Oxygen by Magnesium Oxide Powder. *J. Phys. Chem.* **1978**, *82*, 1028–1032.

(S15) Paukshtis, E. A.; Soltanov, R. I.; Yurchenko, E. N. Determination of the Strength of Aprotic Acidic Centers on Catalyst Surfaces from the IR Spectra of Adsorbed Carbon Monoxide. *React. Kinet. Catal. Lett.* **1981**, *16*, 93–96.

(S16) Wichtendahl, R.; Rodriguez-Rodrigo, M.; Härtel, U.; Kuhlenbeck, H.; Freund, H.-J. Thermodesorption of CO and NO from Vacuum-Cleaved NiO(100) and MgO(100). *Phys. Status Solidi A* **1999**, *173*, 93–100.

(S17) Dohnálek, Z.; Kimmel, G. A.; Joyce, S. A.; Ayotte, P.; Smith, R. S.; Kay, B. D. Physisorption of CO on the MgO(100) Surface. *J. Phys. Chem. B* **2001**, *105*, 3747–3751.

(S18) Henry, C. R.; Chapon, C.; Duriez, C. Precursor State in the Chemisorption of CO on Supported Palladium Clusters. *J. Chem. Phys.* **1991**, *95*, 700–705.

(S19) Jian-Wei, H.; Estrada, C. A.; Corneille, J. S.; Ming-Cheng, W.; Wayne Goodman, D. CO Adsorption on Ultrathin MgO Films Grown on a Mo(100) Surface: An IRAS Study. *Surf. Sci.* **1992**, *261*, 164–170.

(S20) Spoto, G.; Gribov, E.; Damin, A.; Ricchiardi, G.; Zecchina, A. The IR Spectra of ${Mg_5}^{2+}$(CO) Complexes on the (001) Surfaces of Polycrystalline and Single Crystal MgO. *Surf. Sci.* **2003**, *540*, L605–L610.

(S21) Spoto, G.; Gribov, E. N.; Ricchiardi, G.; Damin, A.; Scarano, D.; Bordiga, S.; Lamberti, C.; Zecchina, A. Carbon Monoxide MgO from Dispersed Solids to Single Crystals: A Review and New Advances. *Prog. Surf. Sci.* **2004**, *76*, 71–146.

(S22) Sterrer, M.; Risse, T.; Freund, H.-J. CO Adsorption on the Surface of MgO(001) Thin Films. *Appl. Catal., A* **2006**, *307*, 58–61.

(S23) Grimme, S. Semiempirical GGA-type density functional constructed with a long-range dispersion correction. *J. Comp. Chem.* **2006**, *27*, 1787–1799.

(S24) Tosoni, S.; Sauer, J. Accurate Quantum Chemical Energies for the Interaction of Hydrocarbons with Oxide Surfaces: CH_4/MgO(001). *Phys. Chem. Chem. Phys.* **2010**, *12*, 14330–14340.

(S25) Lazarov, V. K.; Plass, R.; Poon, H.-C.; Saldin, D. K.; Weinert, M.; Chambers, S. A.; Gajdardziska-Josifovska, M. Structure of the Hydrogen-Stabilized MgO}(111)-(1×1) Polar Surface: Integrated Experimental and Theoretical Studies. *Phys. Rev. B* **2005**, *71*, 115434.

(S26) Fuentealba, P.; von Szentpaly, L.; Preuss, H.; Stoll, H. Pseudopotential Calculations for Alkaline-Earth Atoms. *J. Phys. B: Atom. Mol. Phys.* **1985**, *18*, 1287.

(S27) Lu, Y.; Farrow, M. R.; Fayon, P.; Logsdail, A. J.; Sokol, A. A.; Catlow, C. R. A.; Sherwood, P.; Keal, T. W. Open-Source, Python-Based Redevelopment of the ChemShell Multiscale QM/MM Environment. *J. Chem. Theory Comput.* **2019**, 12.

(S28) Shi, B. X.; Kapil, V.; Zen, A.; Chen, J.; Alavi, A.; Michaelides, A. General Embedded Cluster Protocol for Accurate Modeling of Oxygen Vacancies in Metal-Oxides. *J. Chem. Phys.* **2022**, *156*, 124704.

(S29) Nagy, P. R.; Kállay, M. Optimization of the Linear-Scaling Local Natural Orbital CCSD(T) Method: Redundancy-Free Triples Correction Using Laplace Transform. *J. Chem. Phys.* **2017**, *146*, 214106.

(S30) Nagy, P. R.; Samu, G.; Kállay, M. Optimization of the Linear-Scaling Local Natural Orbital CCSD(T) Method: Improved Algorithm and Benchmark Applications. *J. Chem. Theory Comput.* **2018**, *14*, 4193–4215.

(S31) Nagy, P. R.; Kállay, M. Approaching the Basis Set Limit of CCSD(T) Energies for Large Molecules with Local Natural Orbital Coupled-Cluster Methods. *J. Chem. Theory Comput.* **2019**, *15*, 5275–5298.

(S32) Gyevi-Nagy, L.; Kállay, M.; Nagy, P. R. Integral-Direct and Parallel Implementation of the CCSD(T) Method: Algorithmic Developments and Large-Scale Applications. *J. Chem. Theory Comput.* **2020**, *16*, 366–384.

(S33) Nagy, P. R.; Samu, G.; Kállay, M. An Integral-Direct Linear-Scaling Second-Order Møller–Plesset Approach. *J. Chem. Theory Comput.* **2016**, *12*, 4897–4914.

(S34) Kállay, M.; Nagy, P. R.; Mester, D.; Rolik, Z.; Samu, G.; Csontos, J.; Csóka, J.; Szabó, P. B.; Gyevi-Nagy, L.; Hégely, B.; Ladjánszki, I.; Szegedy, L.; Ladóczki, B.; Petrov, K.; Farkas, M.; Mezei, P. D.; Ganyecz, Á. The MRCC Program System: Accurate Quantum Chemistry from Water to Proteins. *J. Chem. Phys.* **2020**, *152*, 074107.

(S35) Kállay, M.; Nagy, P. R.; Mester, D.; Gyevi-Nagy, L.; Csóka, J.; Szabó, P. B.; Rolik, Z.; Samu, G.; Csontos, J.; Hégely, B.; Ganyecz, Á.; Ladjánszki, I.; Szegedy, L.; Ladóczki, B.; Petrov, K.; Farkas, M.; Mezei, P. D.; Horváth, R. A. MRCC*, a quantum chemical program suite. See https://www.mrcc.hu/* **Accessed December 1, 2022**,

(S36) Al-Hamdani, Y. S.; Nagy, P. R.; Zen, A.; Barton, D.; Kállay, M.; Brandenburg, J. G.; Tkatchenko, A. Interactions between Large Molecules Pose a Puzzle for Reference Quantum Mechanical Methods. *Nat. Commun.* **2021**, *12*, 3927.

(S37) Wineman-Fisher, V.; Delgado, J. M.; Nagy, P. R.; Jakobsson, E.; Pandit, S. A.; Varma, S. Transferable Interactions of Li^+ and Mg^{2+} Ions in Polarizable Models. *J. Chem. Phys.* **2020**, *153*, 104113.

(S38) Peterson, K. A.; Dunning, T. H. Accurate Correlation Consistent Basis Sets for Molecular Core–Valence Correlation Effects: The Second Row Atoms Al–Ar, and the First Row Atoms B–Ne Revisited. *J. Chem. Phys.* **2002**, *117*, 10548–10560.

(S39) Balabanov, N. B.; Peterson, K. A. Systematically Convergent Basis Sets for Transition Metals. I. All-electron Correlation Consistent Basis Sets for the 3d Elements Sc–Zn. *J. Chem. Phys.* **2005**, *123*, 064107.

(S40) Kendall, R. A.; Dunning, T. H.; Harrison, R. J. Electron Affinities of the First-row Atoms Revisited. Systematic Basis Sets and Wave Functions. *J. Chem. Phys.* **1992**, *96*, 6796–6806.

(S41) Weigend, F.; Häser, M.; Patzelt, H.; Ahlrichs, R. RI-MP2: Optimized Auxiliary Basis Sets and Demonstration of Efficiency. *Chem. Phys. Lett.* **1998**, *294*, 143–152.

(S42) Hellweg, A.; Hättig, C.; Höfener, S.; Klopper, W. Optimized Accurate Auxiliary Basis Sets for RI-MP2 and RI-CC2 Calculations for the Atoms Rb to Rn. *Theor. Chem. Acc.* **2007**, *117*, 587–597.

(S43) Stoychev, G. L.; Auer, A. A.; Neese, F. Automatic Generation of Auxiliary Basis Sets. *J. Chem. Theory Comput.* **2017**, *13*, 554–562.

(S44) Lehtola, S. Straightforward and Accurate Automatic Auxiliary Basis Set Generation for Molecular Calculations with Atomic Orbital Basis Sets. *J. Chem. Theory Comput.* **2021**, *17*, 6886–6900.

(S45) Neese, F.; Valeev, E. F. Revisiting the Atomic Natural Orbital Approach for Basis Sets: Robust Systematic Basis Sets for Explicitly Correlated and Conventional Correlated *Ab Initio* Methods? *J. Chem. Theory Comput.* **2011**, *7*, 33–43.

(S46) cc4s; available from `https://manuals.cc4s.org`. https://manuals.cc4s.org.

(S47) Kresse, G.; Furthmüller, J. Efficiency of Ab-Initio Total Energy Calculations for Metals and Semiconductors Using a Plane-Wave Basis Set. *Comput. Mater. Sci.* **1996**, *6*, 15–50.

(S48) Grüneis, A.; Booth, G. H.; Marsman, M.; Spencer, J.; Alavi, A.; Kresse, G. Natural Orbitals for Wave Function Based Correlated Calculations Using a Plane Wave Basis Set. *Journal of Chemical Theory and Computation* **2011**, *7*, 2780–2785, PMID: 26605469.

(S49) Hummel, F.; Tsatsoulis, T.; Grüneis, A. Low Rank Factorization of the Coulomb Integrals for Periodic Coupled Cluster Theory. *J. Chem. Phys.* **2017**, *146*, 124105.

(S50) Gruber, T.; Liao, K.; Tsatsoulis, T.; Hummel, F.; Grüneis, A. Applying the Coupled-Cluster Ansatz to Solids and Surfaces in the Thermodynamic Limit. *Phys. Rev. X* **2018**, *8*, 021043.

(S51) Irmler, A.; Gallo, A.; Grüneis, A. Focal-Point Approach with Pair-Specific Cusp Correction for Coupled-Cluster Theory. *J. Chem. Phys.* **2021**, *154*, 234103.

(S52) Schäfer, T.; Gallo, A.; Irmler, A.; Hummel, F.; Grüneis, A. Surface Science Using Coupled Cluster Theory via Local Wannier Functions and In-RPA-embedding: The Case of Water on Graphitic Carbon Nitride. *J. Chem. Phys.* **2021**, *155*, 244103.

(S53) Needs, R. J.; Towler, M. D.; Drummond, N. D.; López Ríos, P.; Trail, J. R. Variational and Diffusion Quantum Monte Carlo Calculations with the CASINO Code. *J. Chem. Phys.* **2020**, *152*, 154106.

(S54) Bennett, M. C.; Melton, C. A.; Annaberdiyev, A.; Wang, G.; Shulenburger, L.; Mitas, L. A New Generation of Effective Core Potentials for Correlated Calculations. *J. Chem. Phys.* **2017**, *147*, 224106.

(S55) Bennett, M. C.; Wang, G.; Annaberdiyev, A.; Melton, C. A.; Shulenburger, L.; Mitas, L. A New Generation of Effective Core Potentials from Correlated Calculations: 2nd Row Elements. *J. Chem. Phys.* **2018**, *149*, 104108.

(S56) Giannozzi, P.; Baroni, S.; Bonini, N.; Calandra, M.; Car, R.; Cavazzoni, C.; Ceresoli, D.; Chiarotti, G. L.; Cococcioni, M.; Dabo, I.; Corso, A. D.; de Gironcoli, S.; Fabris, S.; Fratesi, G.; Gebauer, R.; Gerstmann, U.; Gougoussis, C.; Kokalj, A.; Lazzeri, M.; Martin-Samos, L.; Marzari, N.; Mauri, F.; Mazzarello, R.; Paolini, S.; Pasquarello, A.; Paulatto, L.; Sbraccia, C.; Scandolo, S.; Sclauzero, G.; Seitsonen, A. P.; Smogunov, A.; Umari, P.; Wentzcovitch, R. M. QUANTUM ESPRESSO: A Modular and Open-Source Software Project for Quantum Simulations of Materials. *J. Phys.: Condens. Matter* **2009**, *21*, 395502.

(S57) Fraser, L. M.; Foulkes, W. M. C.; Rajagopal, G.; Needs, R. J.; Kenny, S. D.; Williamson, A. J. Finite-Size Effects and Coulomb Interactions in Quantum Monte Carlo Calculations for Homogeneous Systems with Periodic Boundary Conditions. *Phys. Rev. B* **1996**, *53*, 1814–1832.

(S58) Williamson, A. J.; Rajagopal, G.; Needs, R. J.; Fraser, L. M.; Foulkes, W. M. C.;

Wang, Y.; Chou, M.-Y. Elimination of Coulomb Finite-Size Effects in Quantum Many-Body Simulations. *Phys. Rev. B* **1997**, *55*, R4851–R4854.

(S59) Kent, P. R. C.; Hood, R. Q.; Williamson, A. J.; Needs, R. J.; Foulkes, W. M. C.; Rajagopal, G. Finite-Size Errors in Quantum Many-Body Simulations of Extended Systems. *Phys. Rev. B* **1999**, *59*, 1917–1929.

(S60) Kresse, G.; Furthmüller, J. Efficient Iterative Schemes for Ab Initio Total-Energy Calculations Using a Plane-Wave Basis Set. *Phys. Rev. B* **1996**, *54*, 11169–11186.

(S61) Pople, J. A. Quantum Chemical Models (Nobel Lecture). *Angew. Chem., Int. Ed.* **1999**, *38*, 1894–1902.

(S62) Zen, A.; Brandenburg, J. G.; Klimeš, J.; Tkatchenko, A.; Alfè, D.; Michaelides, A. Fast and Accurate Quantum Monte Carlo for Molecular Crystals. *Proc. Natl. Acad. Sci. U. S. A.* **2018**, *115*, 1724–1729.

(S63) Nygren, M. A.; Pettersson, L. G. M. Comparing Ab Initio Computed Energetics with Thermal Experiments in Surface Science: CO/MgO(001). *J. Chem. Phys.* **1996**, *105*, 9339–9348.

(S64) Pacchioni, G. Quantum Chemistry of Oxide Surfaces: From CO Chemisorption to the Identification of the Structure and Nature of Point Defects on MgO. *Surf. Rev. Lett.* **2000**, *07*, 277–306.

(S65) Sauer, J. Ab Initio Calculations for Molecule–Surface Interactions with Chemical Accuracy. *Acc. Chem. Res.* **2019**, *52*, 3502–3510.

(S66) Campbell, C. T.; Sellers, J. R. V. Enthalpies and Entropies of Adsorption on Well-Defined Oxide Surfaces: Experimental Measurements. *Chem. Rev.* **2013**, *113*, 4106–4135.

(S67) Redhead, P. A. Thermal Desorption of Gases. *Vacuum* **1962**, *12*, 203–211.

(S68) Grimme, S. Supramolecular Binding Thermodynamics by Dispersion-Corrected Density Functional Theory. *Chem. - Eur. J.* **2012**, *18*, 9955–9964.

(S69) Li, Y.-P.; Gomes, J.; Mallikarjun Sharada, S.; Bell, A. T.; Head-Gordon, M. Improved Force-Field Parameters for QM/MM Simulations of the Energies of Adsorption for Molecules in Zeolites and a Free Rotor Correction to the Rigid Rotor Harmonic Oscillator Model for Adsorption Enthalpies. *J. Phys. Chem. C* **2015**, *119*, 1840–1850.
